# Supplementary material for: The rhizodynamics robot: Automated imaging system for studying long-term dynamic root growth
Source: PLoS One. 2023 Dec 21;18(12):e0295823. doi: 10.1371/journal.pone.0295823 (PMC10734993; doi:10.1371/journal.pone.0295823)

**Supplement 1**

**Construction of a simple one-shelf imaging robot**

The size and configuration of the imagining robot is easily adaptable. In this supplement we describe the creation of a single shelf robot capable of imaging 10 magenta boxes simultaneously. This simple design employs all of the mechanisms necessary to create larger, mult-shelf robots. The points where this basic blueprint is expandable to a larger number of shelves are highlighted at the end of this protocol.

The basic robot we are aiming to create is shown below:


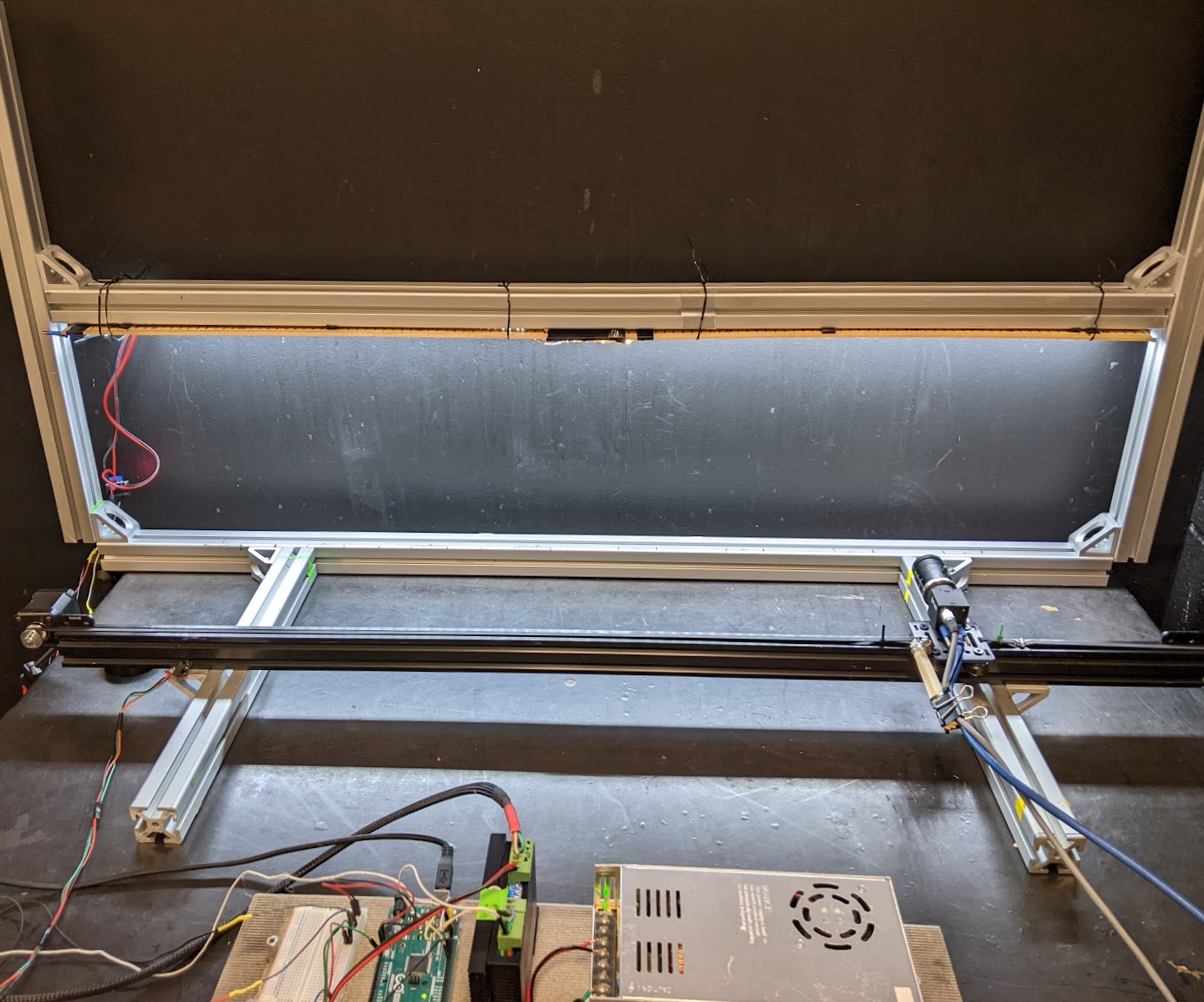


The starting point is assembly of a frame. Here we use 8020 framing metal (this can be cut in the machine shop at most research institutions, or required sizes here are all approximate and standard sizes can be used instead):

48” (2x)

24” (2x)

18” (2x)

Affix the 48” and 24” pieces into a frame using Gussett brackets. This frame will hold the magenta boxes and lights. The distance from the top and bottom horizontal support is adjustable. In this robot we use a distance of 9”.


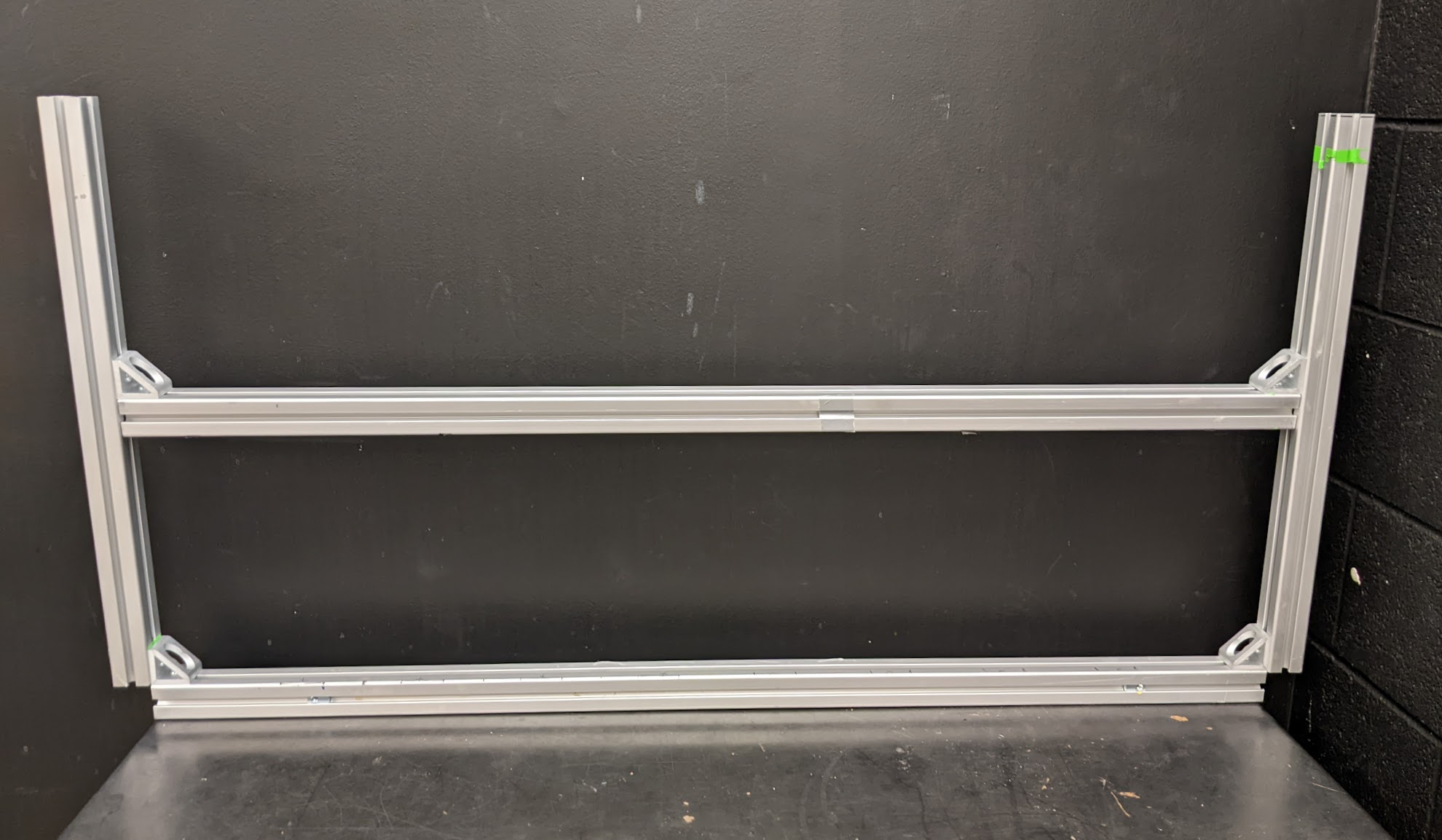


Next, attach the 18” pieces to the base of the rectangular frame using Gussett brackets. Attachment site is not critical but should be approximately 6” in from each end of the frame. These pieces will support the gantry.


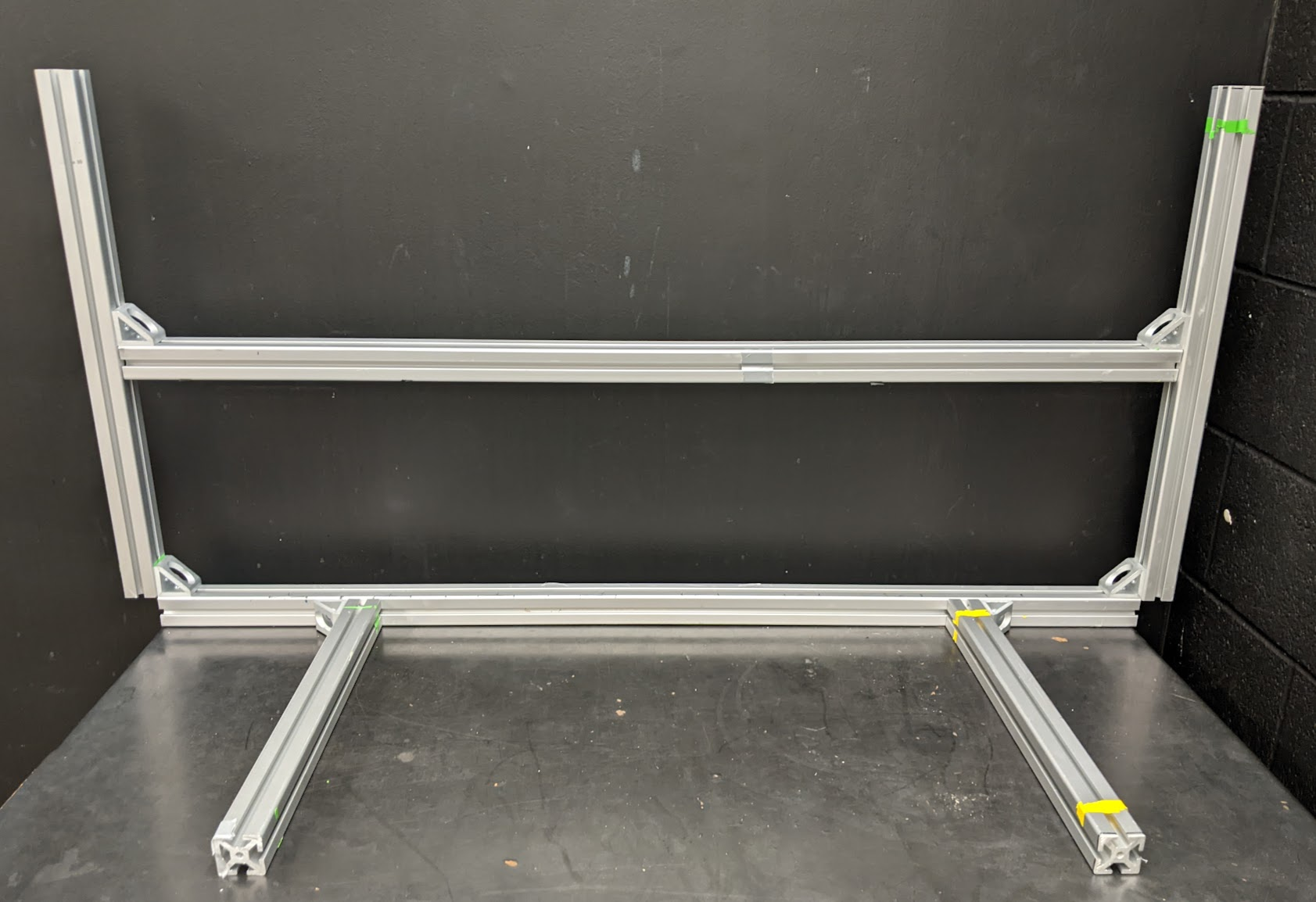


Next assemble the gantry according to manufacturer’s instructions (several excellent tutorials exist on youtube demonstrating the assembly). Add torsion spring to increase tension on the belt. Affix gantry to the 18” 8020 pieces using M5 drop-in fasteners. Attach camera using a tripod screw and install lens. We use a distance of 9.25“ from the lens to the shelf for imaging magenta boxes, but this is flexible based on the desired imaging parameters.


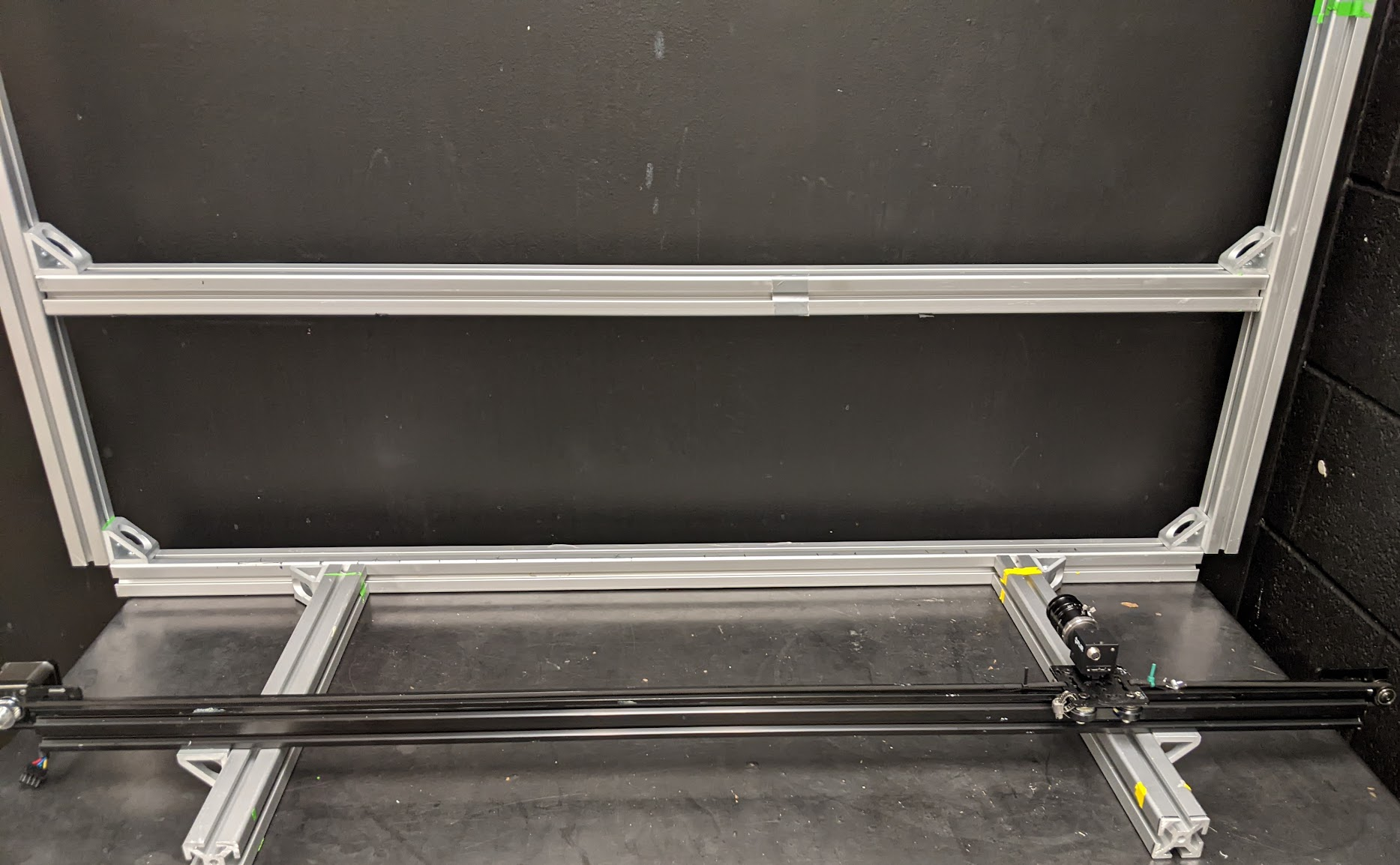


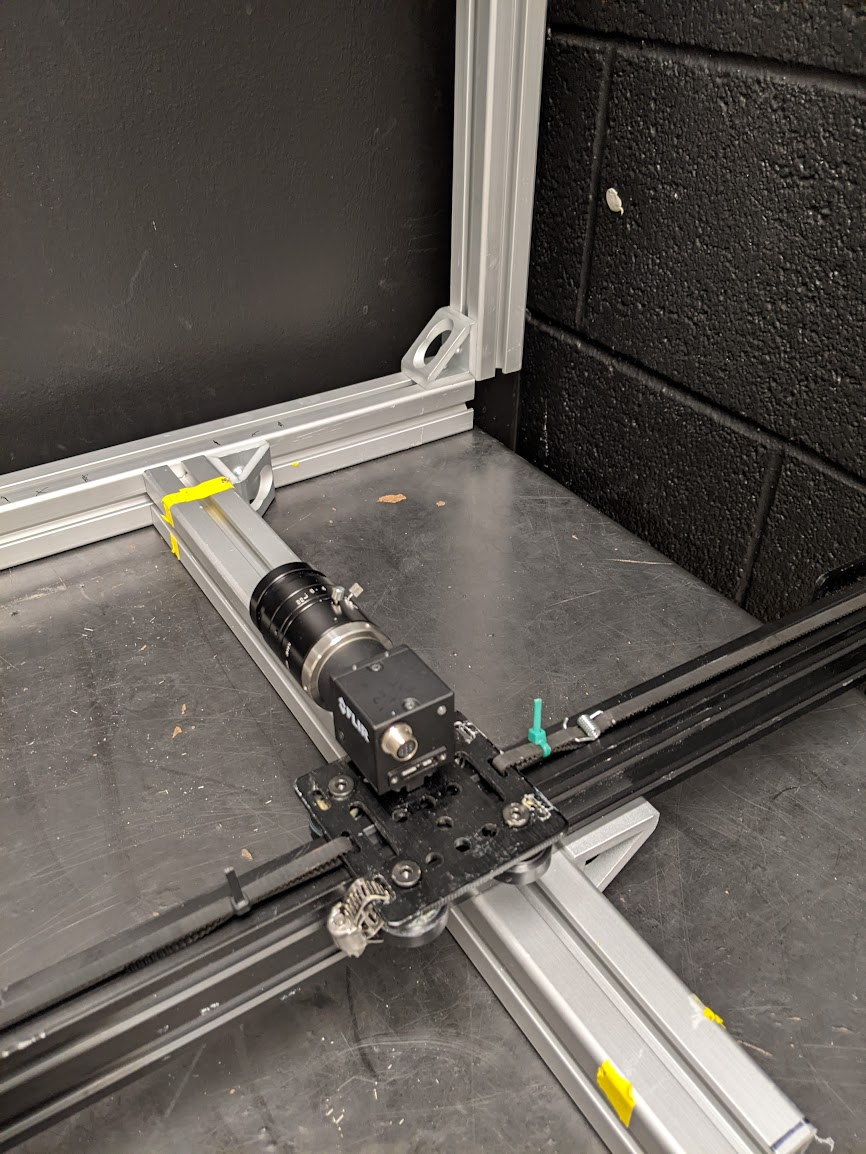


Assemble the light panel. For lack of an obvious alternative we use yard sticks affixed with perpendicular supports by hot glue as the support for LED strips. We have found 6 strips of LEDs at a distance of ~25 cm produces light at an intensity comparable to that produced in our Arabidopsis growth chambers (6800 lux).


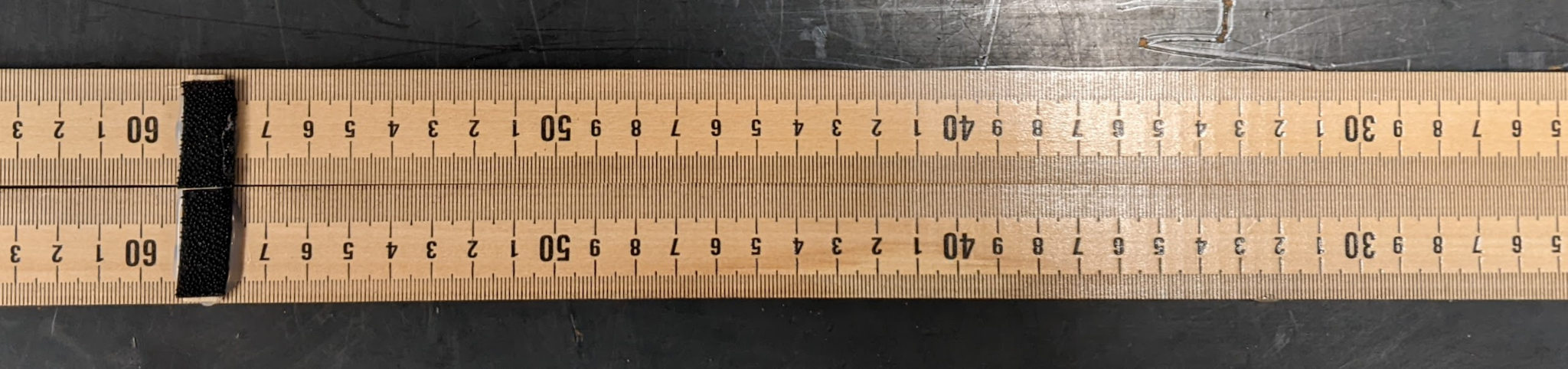


Attach the LED strips in alternating orientations using peel off adhesive, cutting along the prescribed cut lines marked on the stips, and soldering short (3-4 cm 22 guage) connecting wires as needed. Many youtube tutorials discuss soldering LED strips, and we found after a small amout of practice our speed improved dramatically. We cover the soldered points with electricians tape.


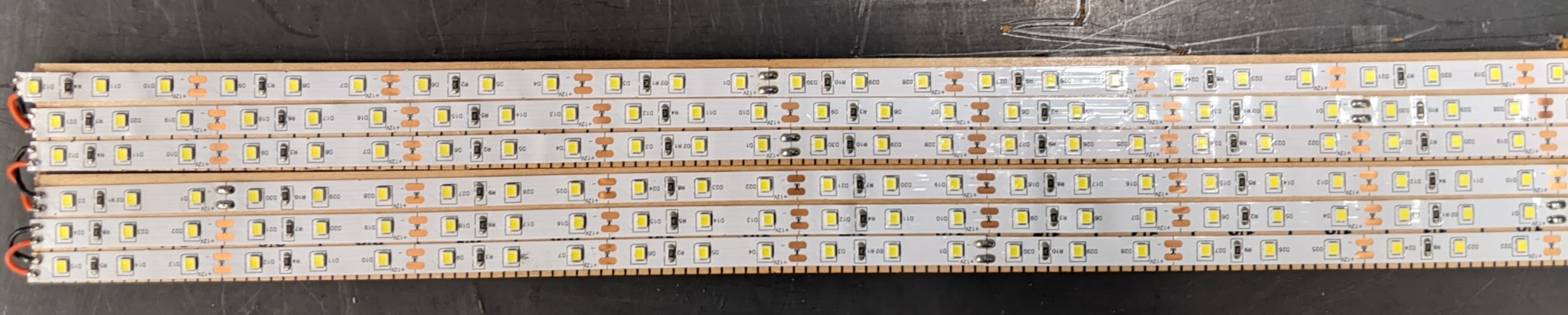


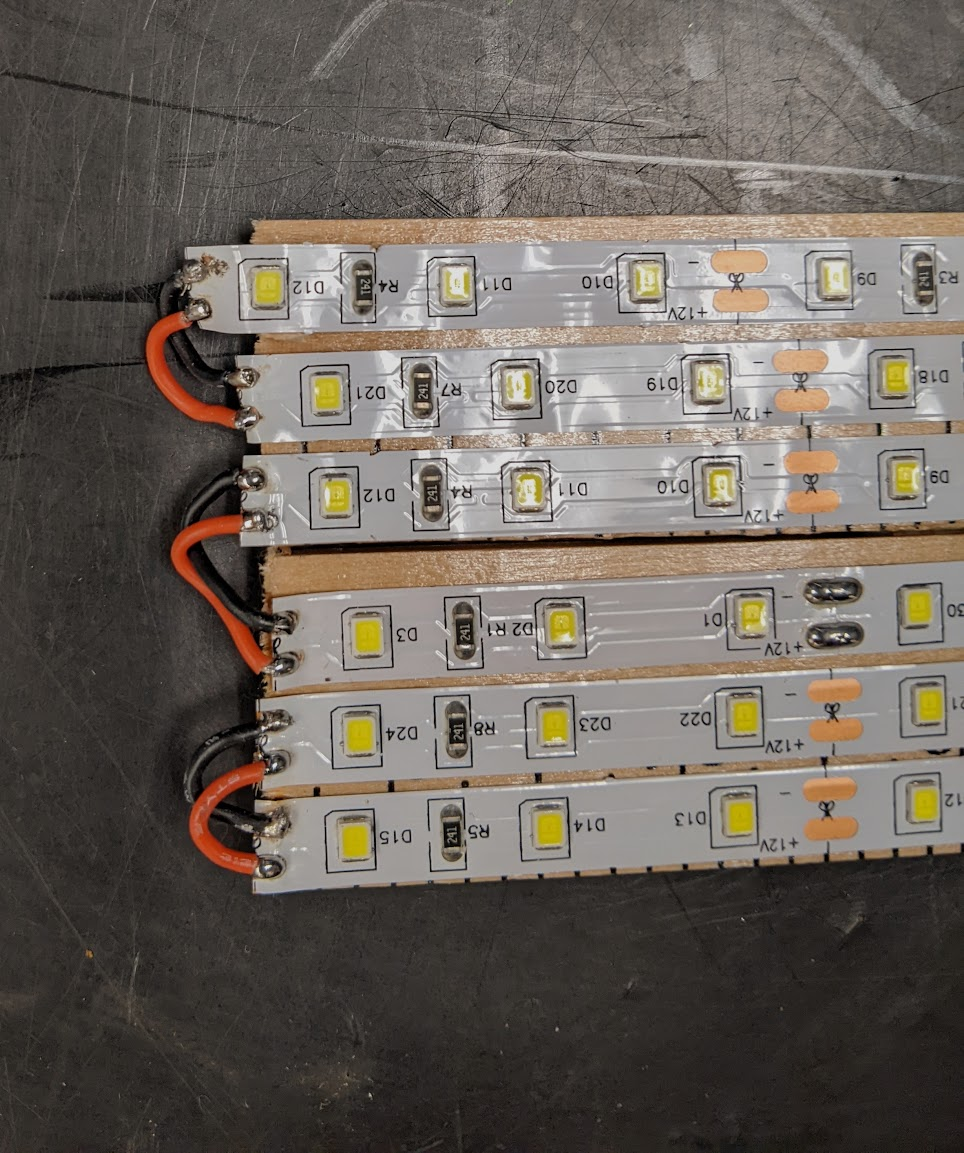


We cut and strip the power supply wires for the LEDs and run through a relay. This will be wired to the Arduino in order to turn on or off the lights (described below). We use the optional dimmer switch that comes with the LEDs in order to be able to adjust light intensity. In our setup the negative wire is run to the LEDs and the positive wire is routed through the relay.


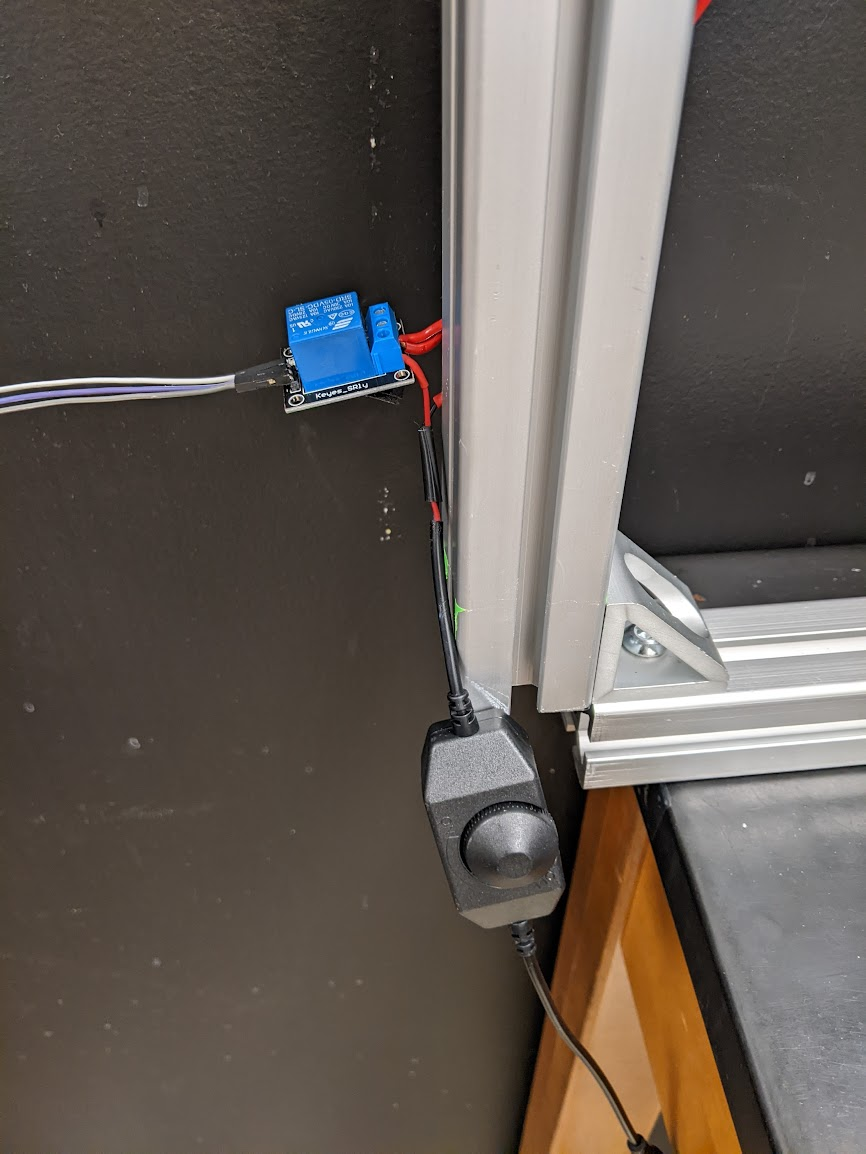


Next we attach the lights to the underside of the top 8020 piece in the frame. We have found twist-tie works well for securing the lights while offering easy removal.


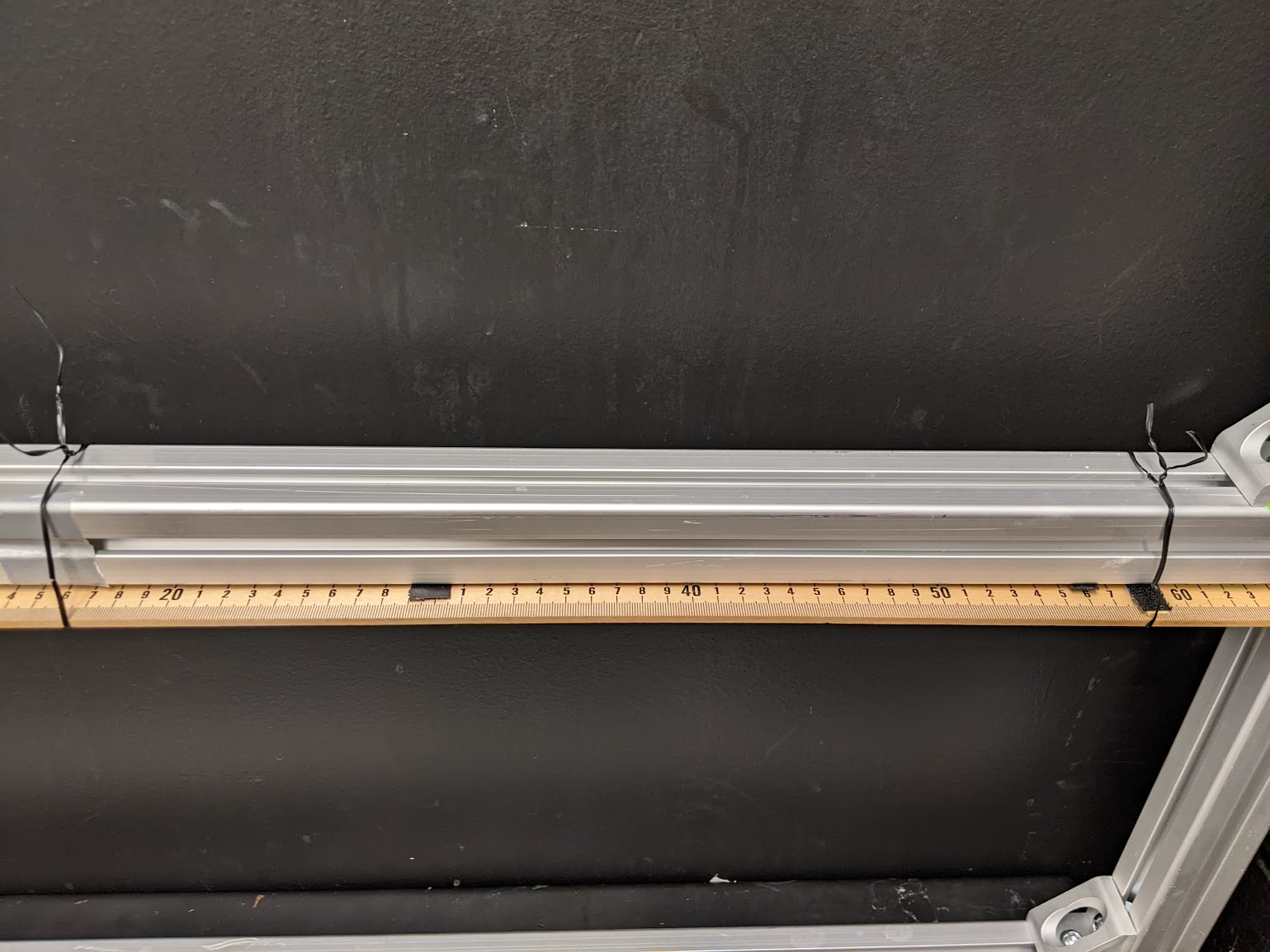


Next we assemble the power supply, stepper motor driver, arduino, and breadboard on a non-conductive surface. We use rubber coated slabs scavenged from a construction site. Once everything is wired we tack the 4 components shown in place with hot glue. Our goal is the configuration shown below.


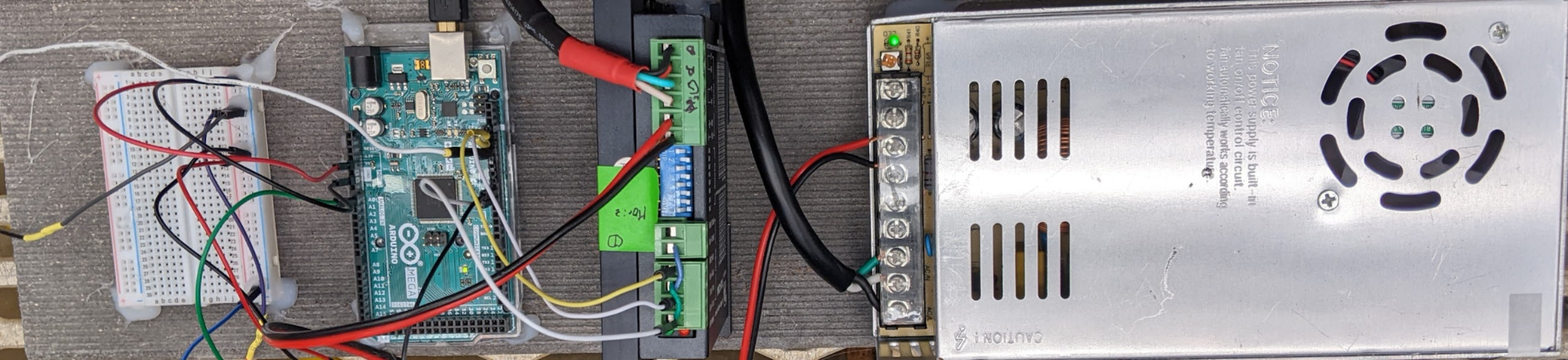


IMPORTANT: Do not plug power supply in until ready for unit testing.

Connect the positive terminal of the power supply to the driver.

First using 22 guage wire, we connect the Stepper control pins as follows:

PUL+ connect to DIR+

DIR+ connect to ENBL+

Next connect three male-male jumper cables to PUL-, DIR-, and ENBL+ (these will also be connected to Arduino described below). A close-up of how the control pins should be wired is shown below (note that this is image is of a different robot from that shown above and the jumper wire colors are different). Tighten all connections with a small flat-head screwdriver.


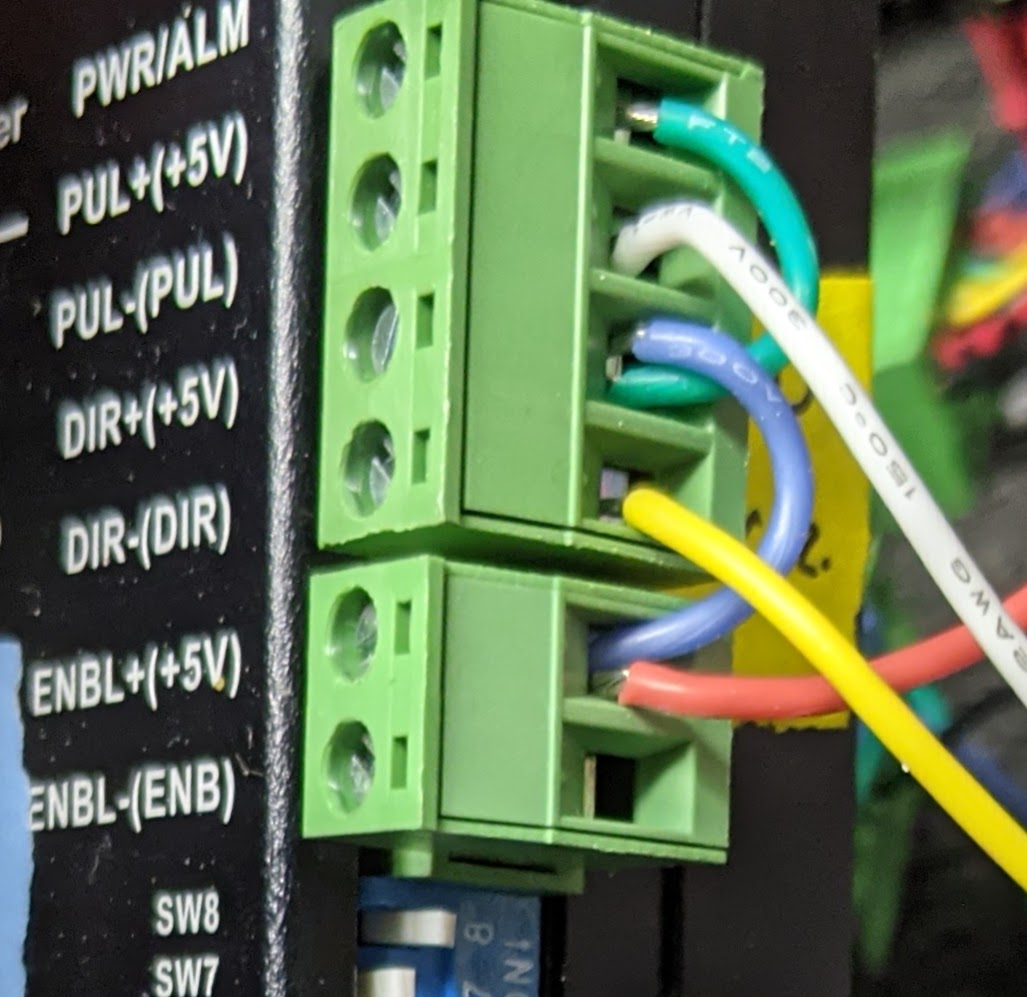


Connect the other ends of the jumper wires to Arduino digitical pins as follows:

ENBL+ connect to D10

DIR- connect to D9

PUL- connect to D8


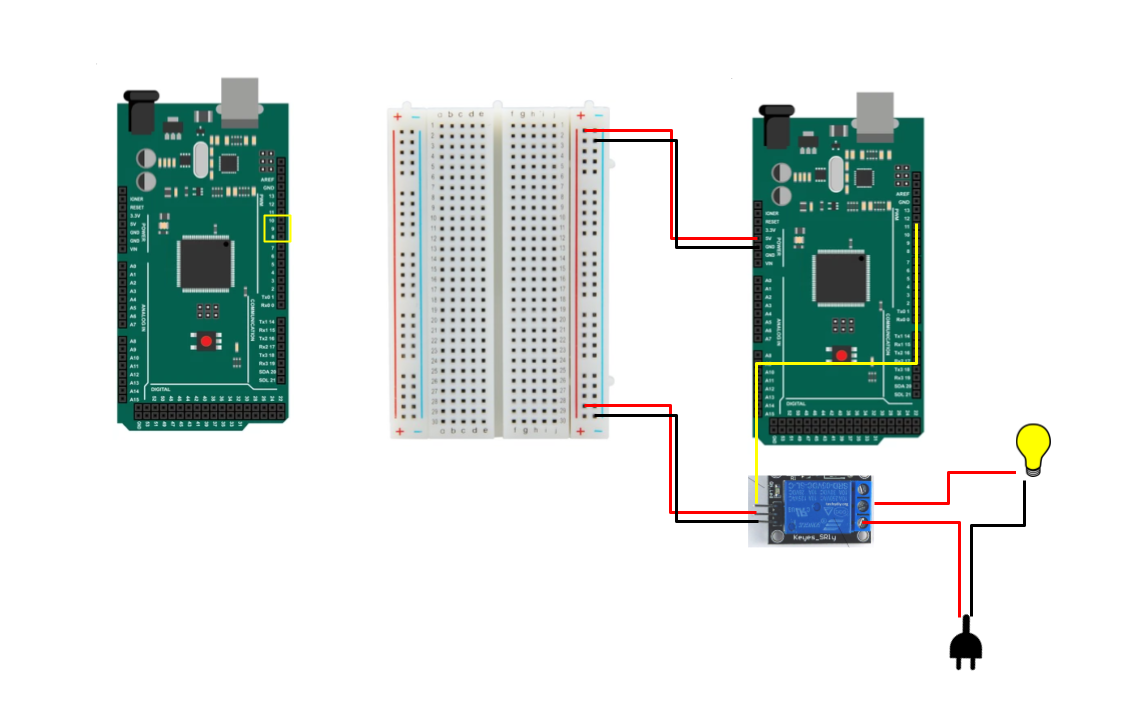


Use following DIP switch configurations for single motor to supply approximately 2 amps current: ON OFF ON OFF ON OFF OFF ON


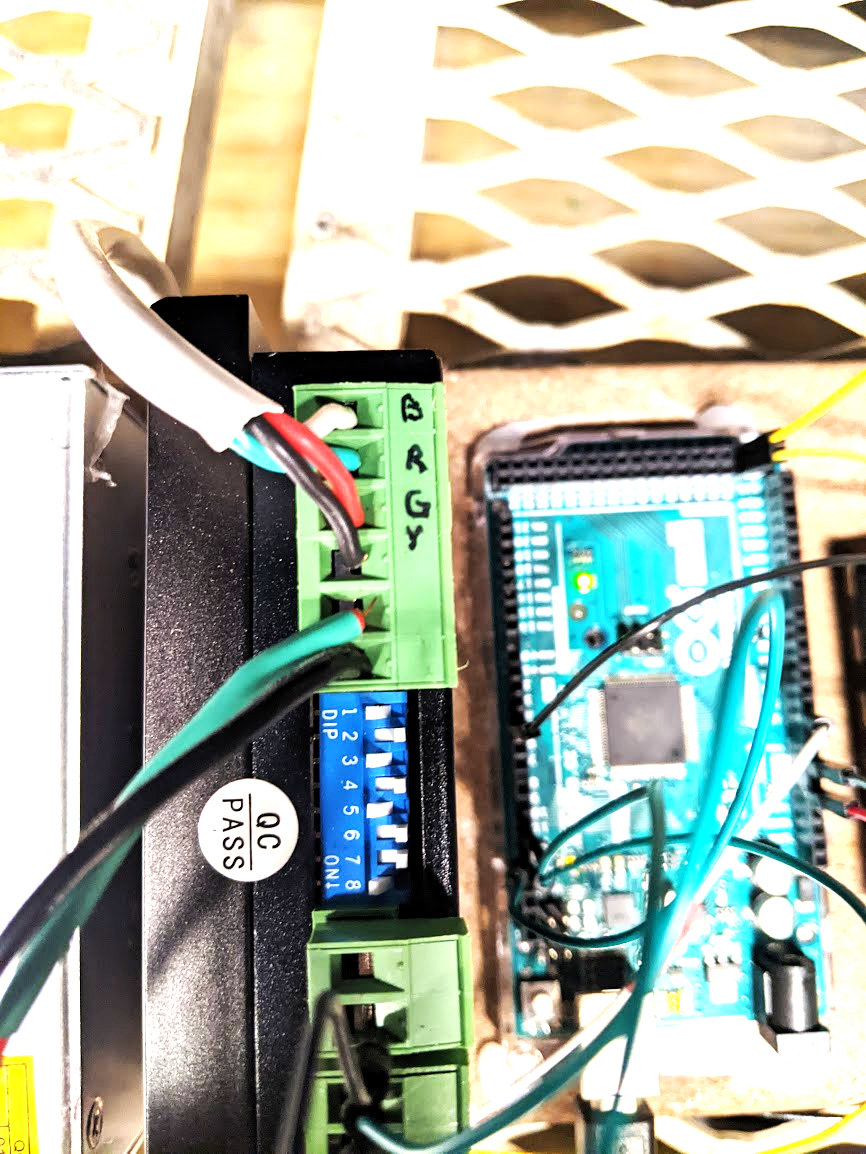


Connect the power supply to the positive, negative, and ground cable of the stripped and soldered ends of the power supply cable, while also connecting 18 guage wire to the positive and negative poles power supply to the DC+ and DC- pins of the driver, respectively.


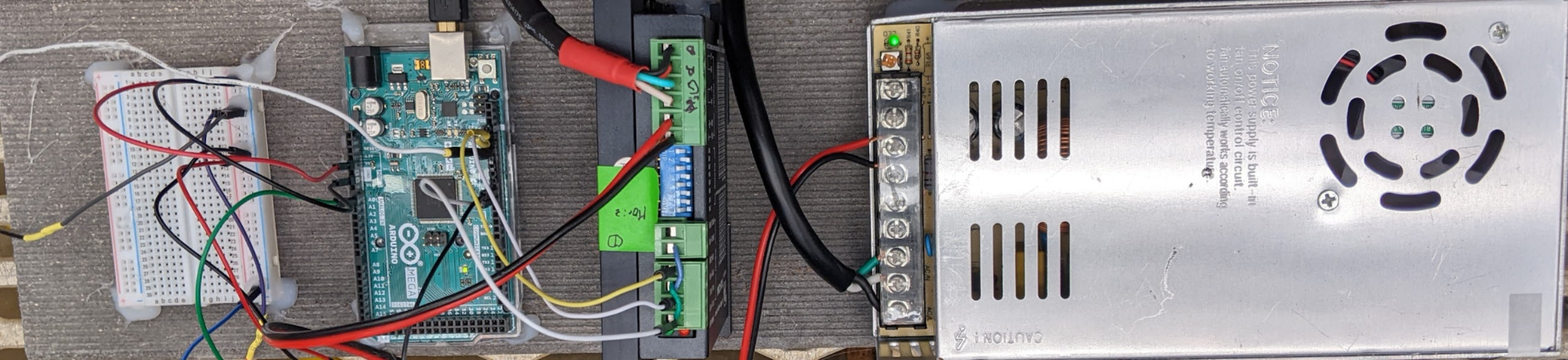

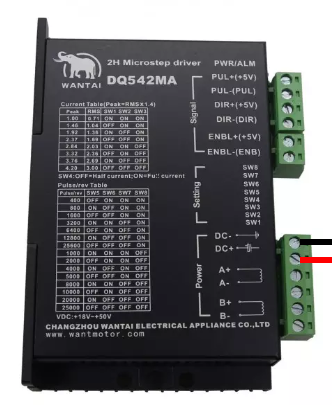


Finally connect the motor to the driver. Connect one of the coils to the A- and A+ pins, and the second to the B- and B+ pins. The extension cables provided with the motors can be stripped and then connected:

YELLOW -> A-

GREEN -> A+

BLUE -> B-

RED -> B


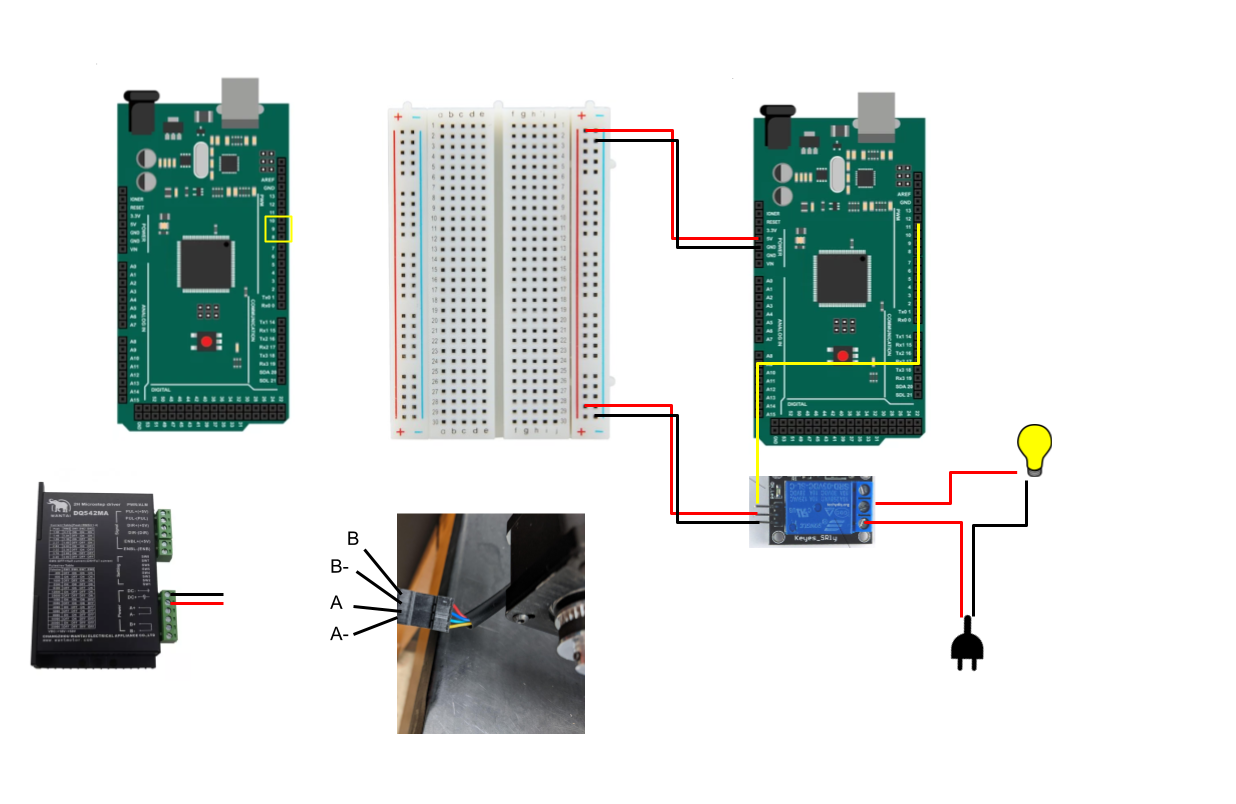


Connect a ground wire from the arduino to the negative rail on the bread board using a male-male jumper cable. Connect the 5V arduino pin to the positive rail of the bread board in the same way. To keep wires secure on the breadboard, we tack them in place with a small bit of hot glue.


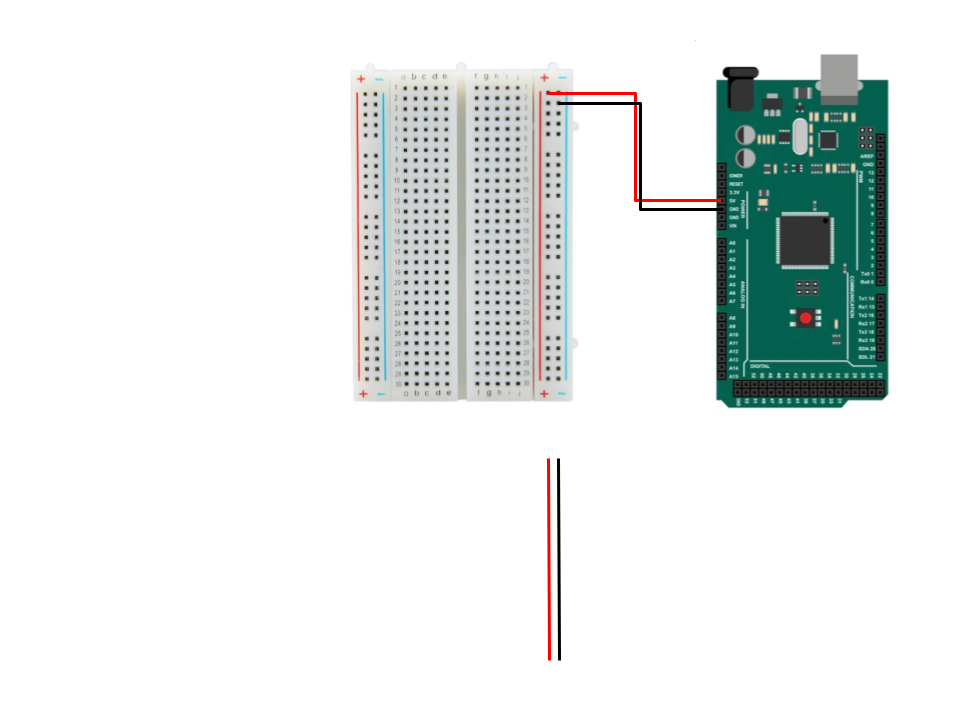


The pins on the relay are labelled “+”, “-”, and “S” (signal). Connect the + and - pins on the relay to the breadboard using male-male jumper cable connections. Connect the signal pin to digital pin D11 on the arduino.


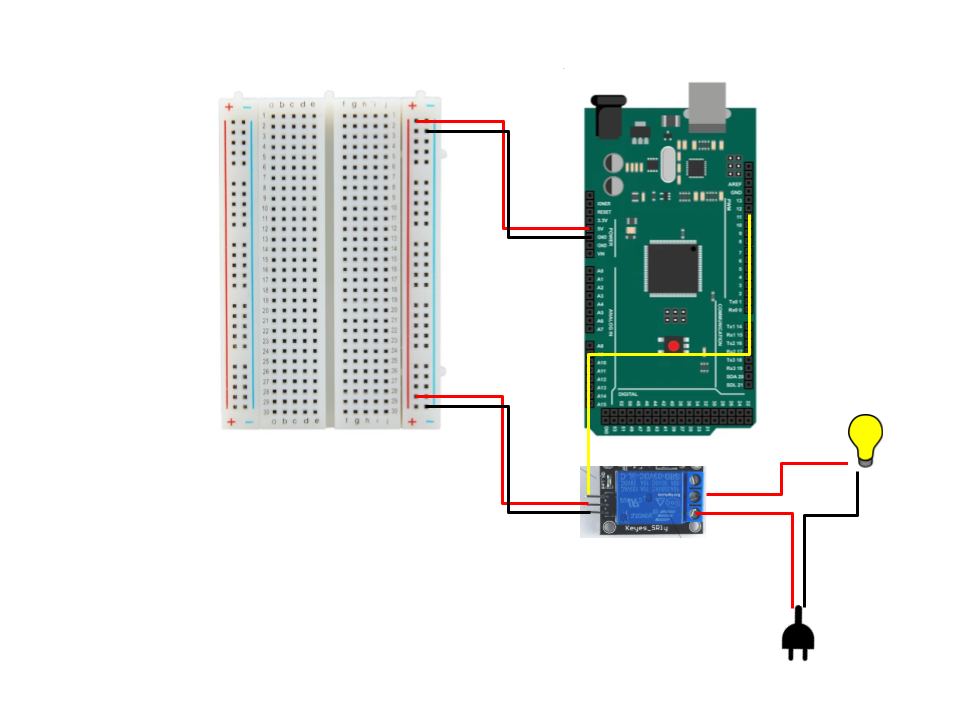


Snap the photointerrupeter into the breakout board and solder the connections along with a 225 ohm resistor and wires to the “PWR”, “GRD”, and “SIG” leads.


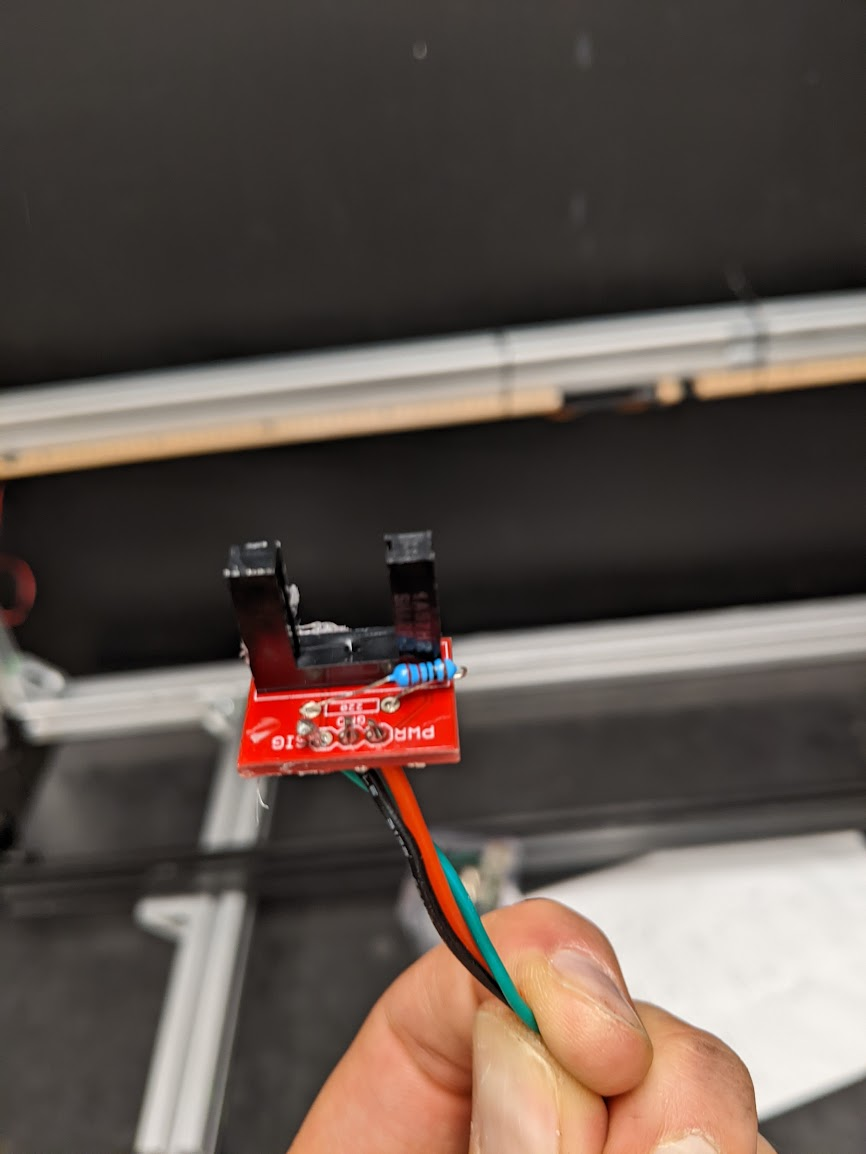


Connect the PWR and GRD wires of the photointerruptor to the 5V and ground rails on the breadboard, and connect the SIG wire to analog pin A0 on the arduino.


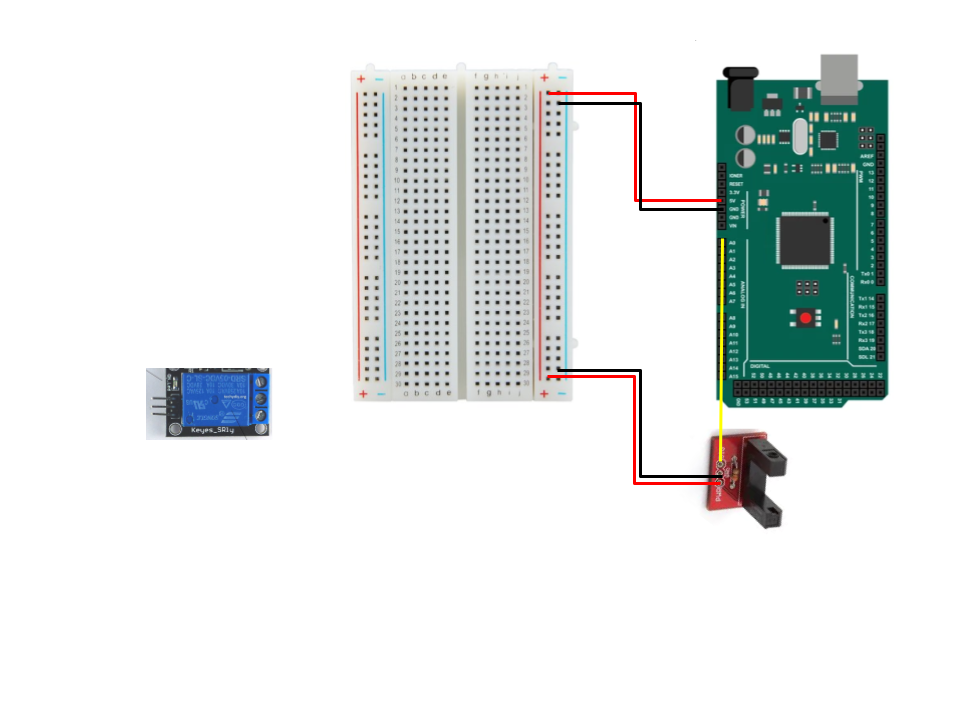


For this robot we use the photointerruptor to define the “home” position for the camera to be just above the left gantry support. This will be the location the camera returns after each imaging cycle. Affix the photo interrupter to the bottom of the gantry rail with hot glut along with a blocker on the bottom of one of the wheels underneath the camera plate. We have found a small piece of think cardboard covered by electrician’s tape works well as a blocker.


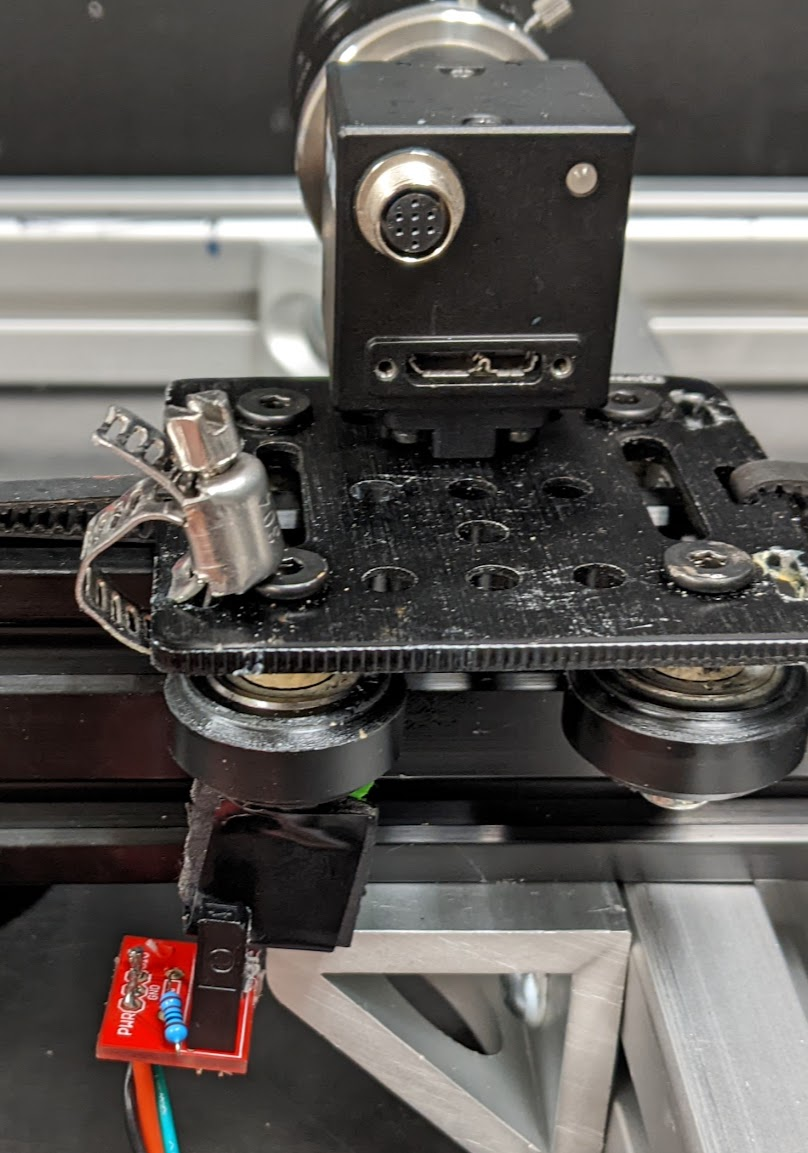


The GPIO cord has a number of wires we won’t use. Connect the blue wire to arduino ground on the bread board, and the black wire to digitial pin D6. We typiclaly strip the wires and solder to another stripped jumper cable, joining with heat shrink.


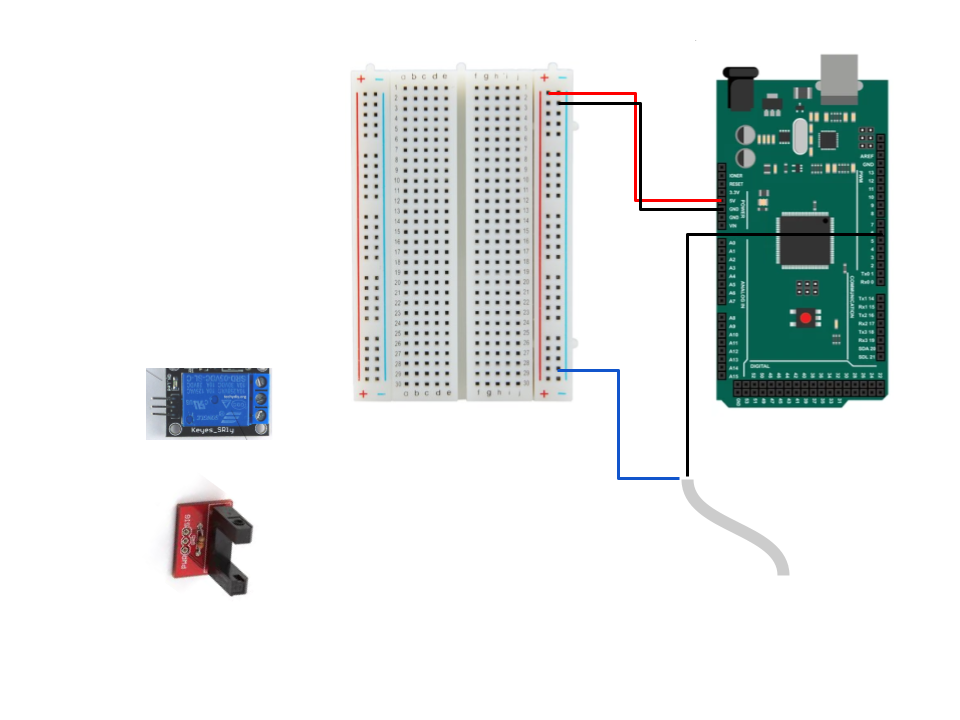


We typically attach a rigid post of some kind to the camera plate using a small hose clamp, and affix the wires to the clamp. This prevents stress on the camera/wire connections. Below we use a few small wooden scrap strips and a binder clip and zip tie.


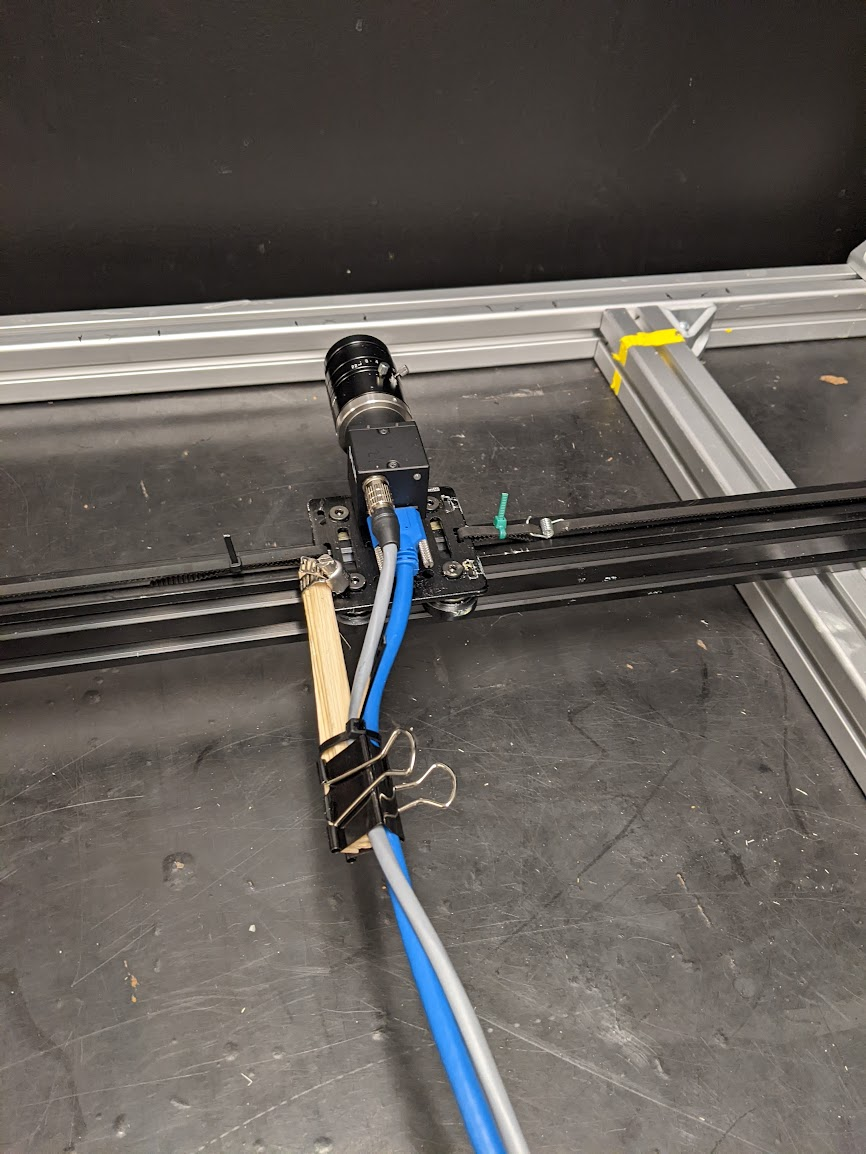


We typically suspend the wires with a string hanging above the robot. The objective is to create a flexible suspension to keep the wires out of the way of the gantry. An example is shown below.


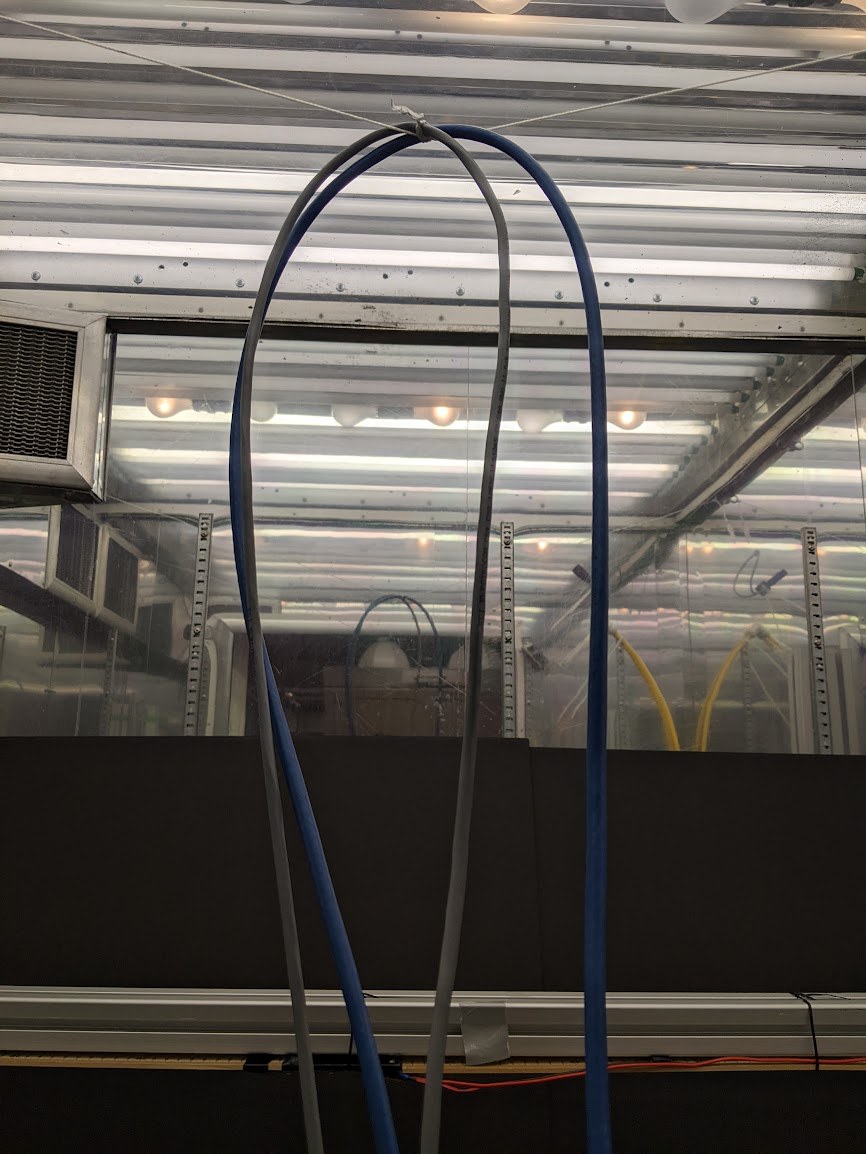


Finally, plug in power supply for stepper driver as well as lights. Connect arduino and the camera to the computer via USB cables. Drape fabric or other light blocking material around robot as needed. We also found attaching a piece of poster board to the lens of the camera using hot glue with a hole cut out reduces glare. Example below.


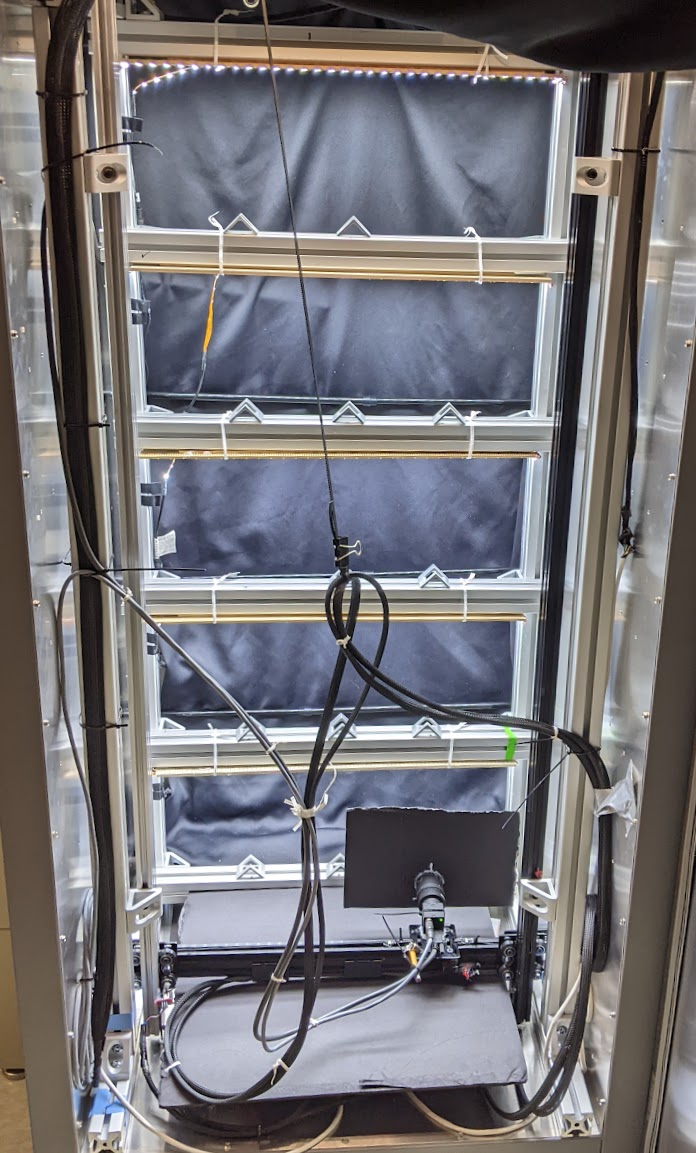


For multi-shelf robots, the assembly is essentially the same. The main difference is that frame must by created as a shelf system which is simply the same system with taller vertical 8020 segments and additional shelves and light banks. An example is shown below.


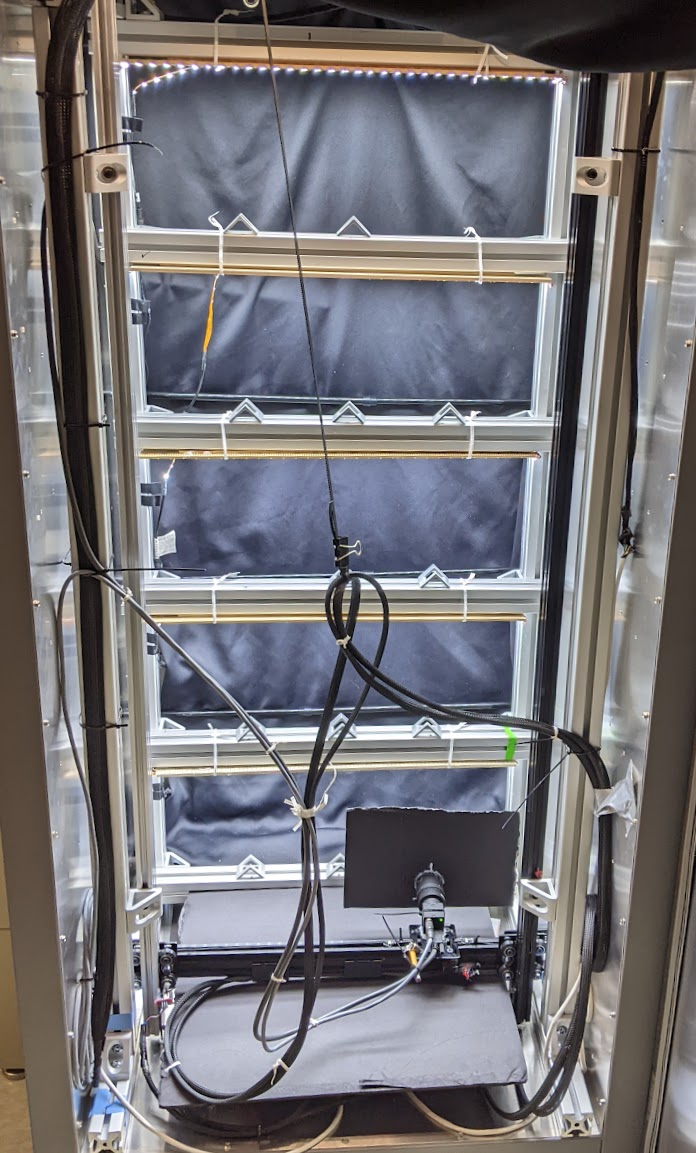


In addition a second motor driver needs to be connected to arduino and the power supplies. We mount the two motors at the top of the shelves.


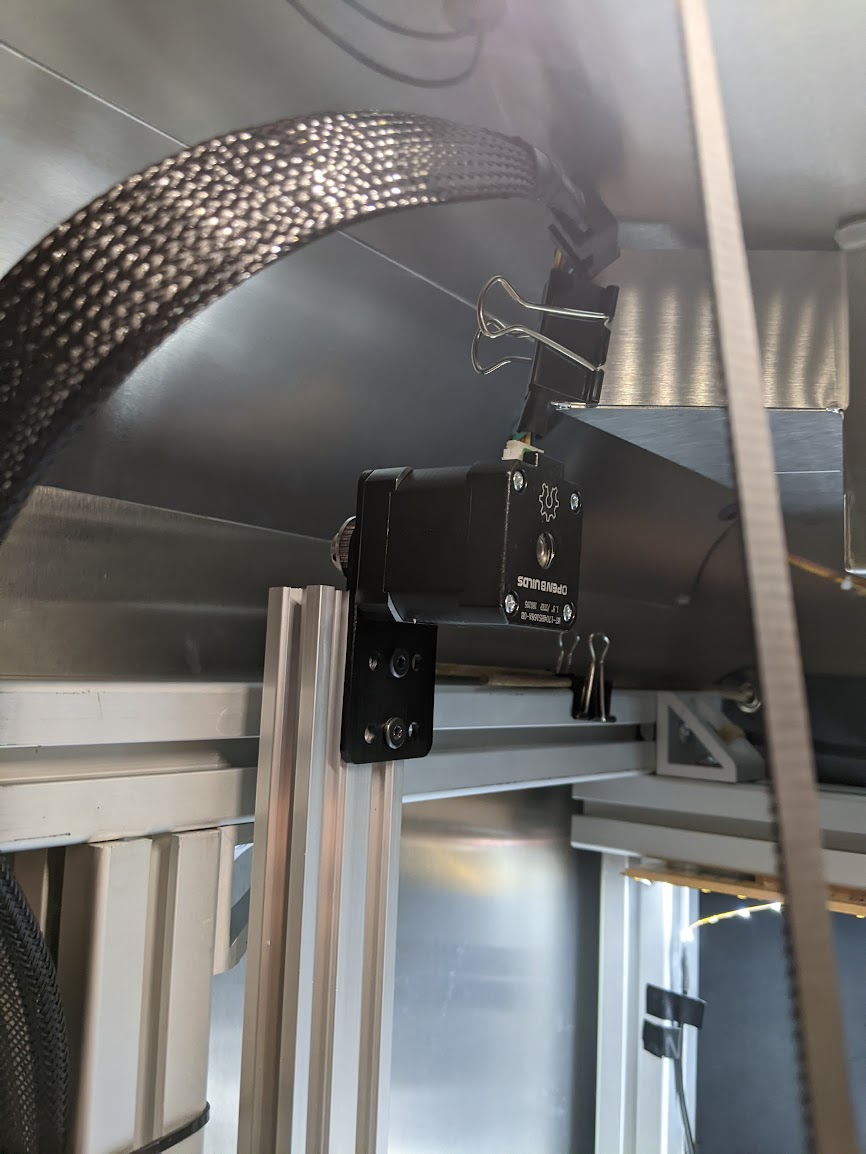

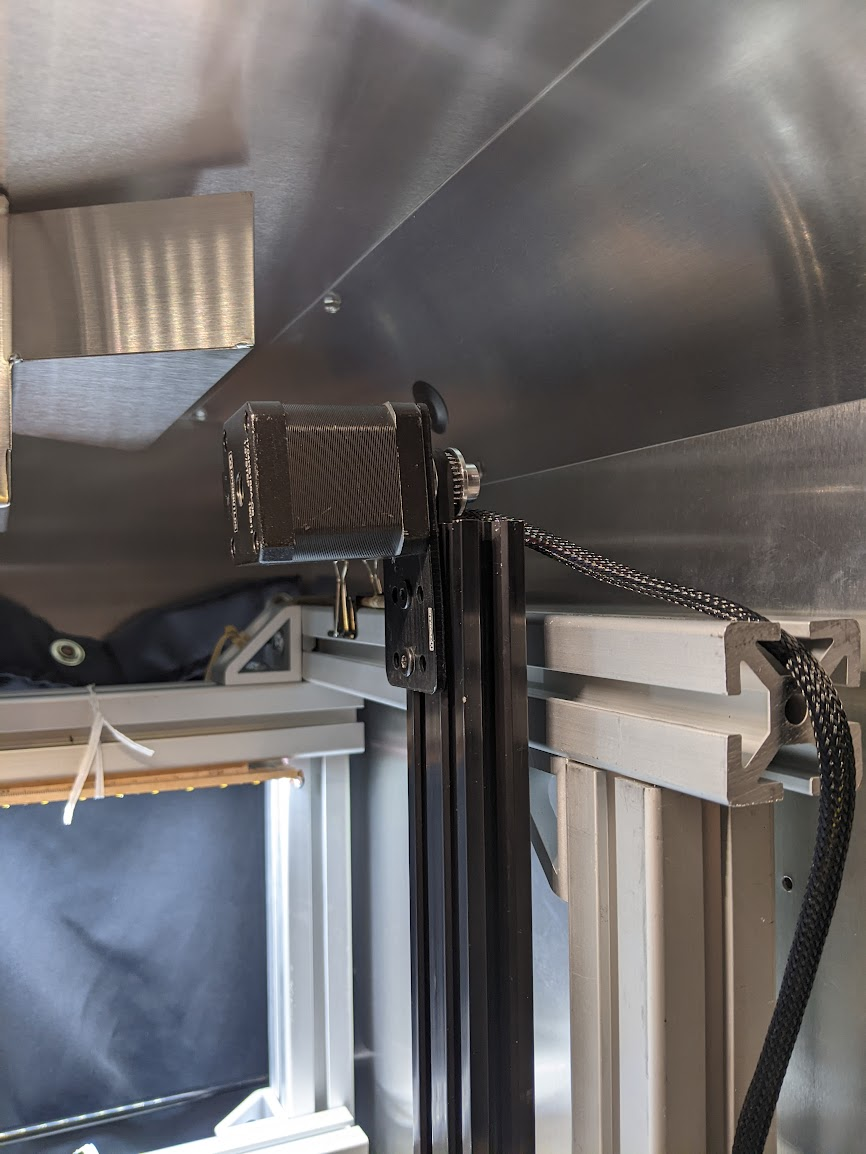


We mount the horizontal gantry to the vertical gantries at both ends.


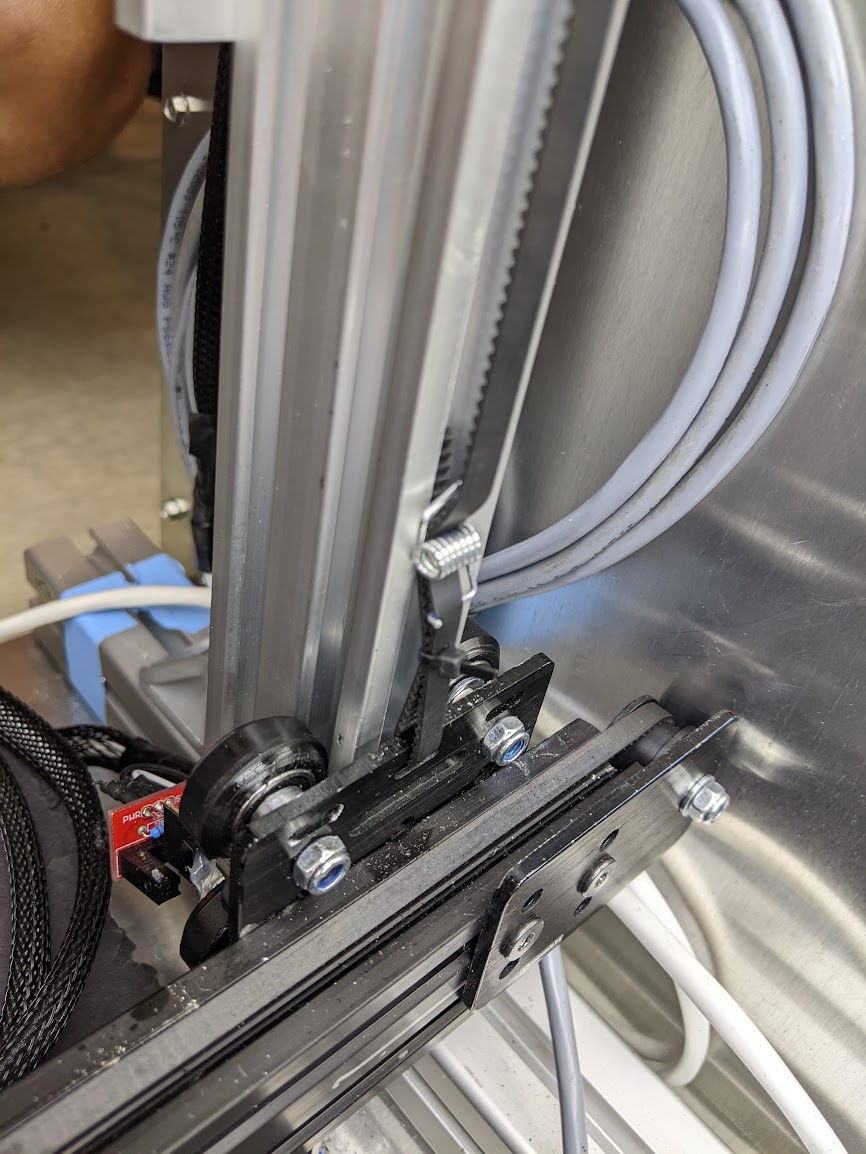

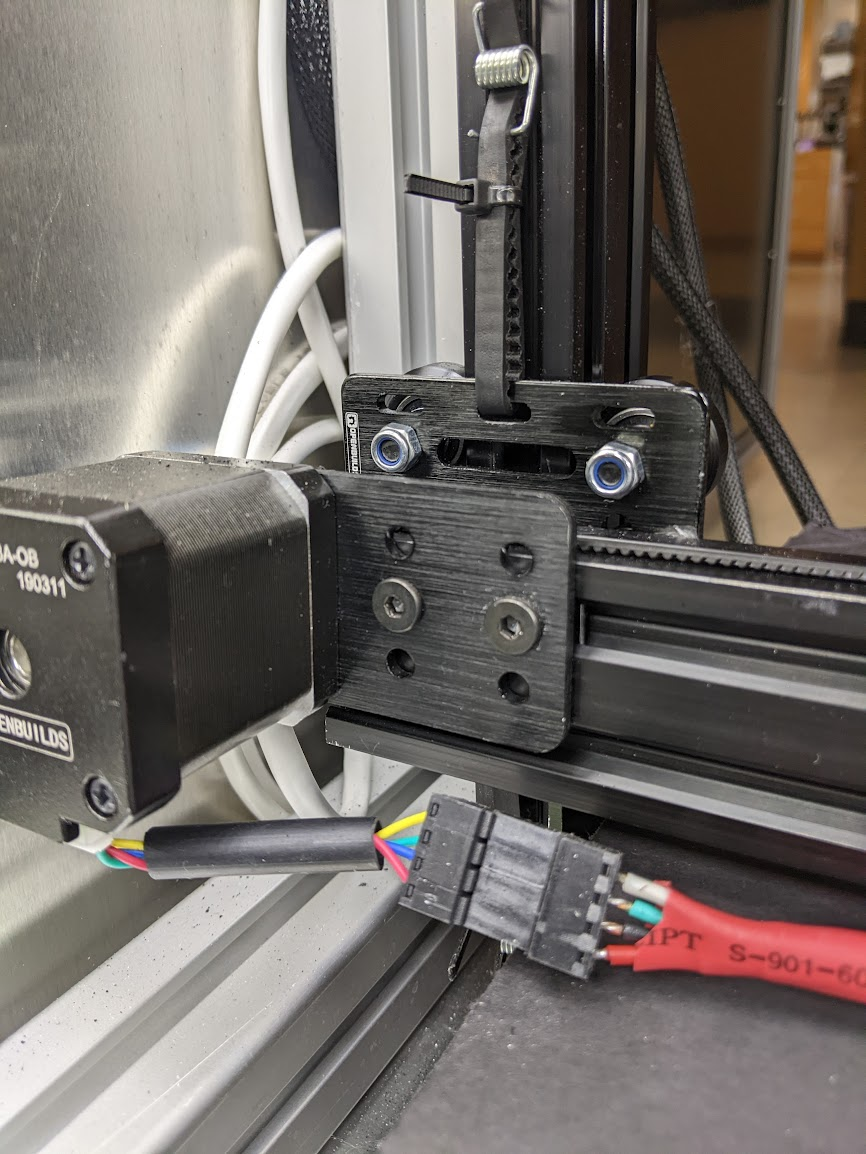


Finally, a second photointerrupter should be placed at the home location such that the vertical plates hit the interrupter.


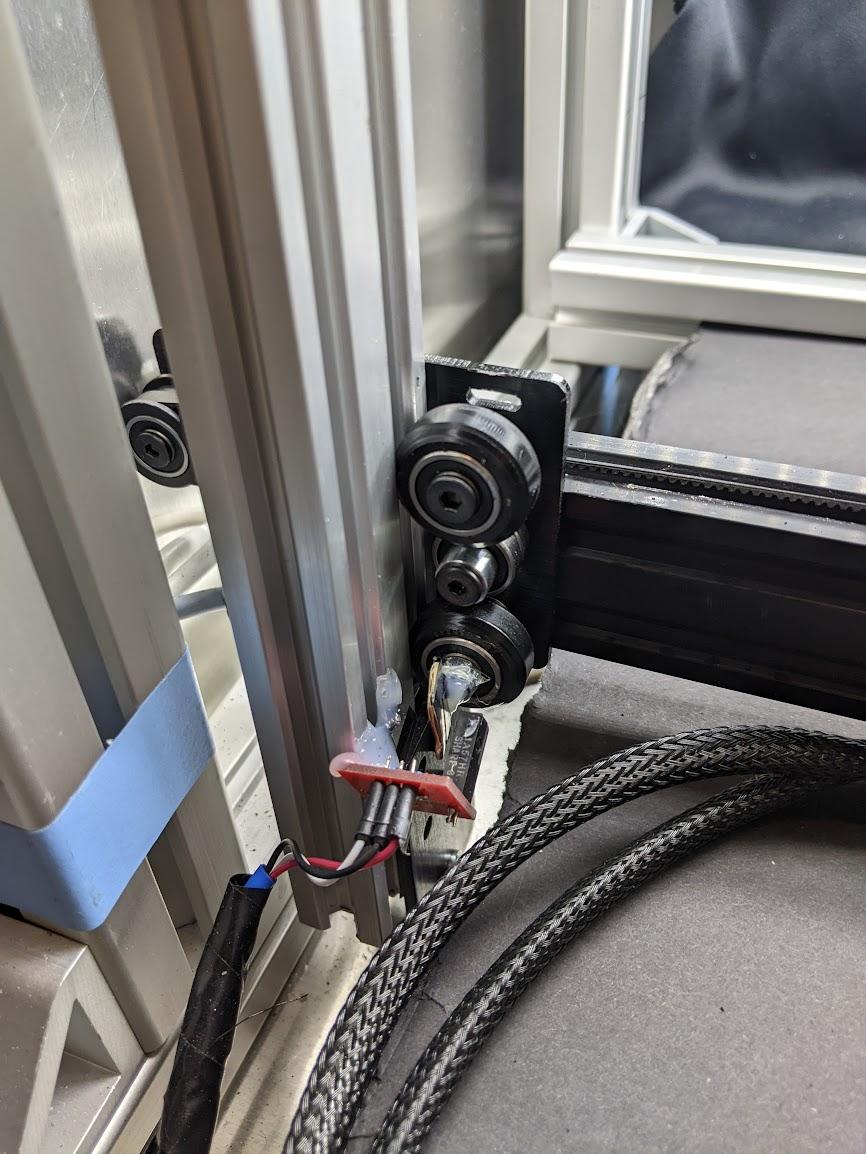

Supplement: S1 File — (DOCX) [file pone.0295823.s001.docx]
