# Supplementary material for: The rhizodynamics robot: Automated imaging system for studying long-term dynamic root growth
Source: PLoS One. 2023 Dec 21;18(12):e0295823. doi: 10.1371/journal.pone.0295823 (PMC10734993; doi:10.1371/journal.pone.0295823)

**Supplement 2**

**Robot control code**

Included here is the code needed to run a simple one-shelf robot as described in Supplement 1. We are happy to provide assistance in helping develop more complicated robot programs. Please contact us via the corresponding author or (even better) on the GROOT GitHub page.

**Hardware and Software requirements:**

We use an AlienWare Desktop running Windows 10. The specifications are not critical because the function of the computer is mainly to load code to the Arduino and to store/transfer images being captured by the camera. Flycapture/Flycap is the program which controls the function of the camera and saves the images. It runs and Windows and Linux. We have only tested this system on Windows. Arduino IDE is the coding environment for writing programs for the Arduino. JupyterLab is a multi-functional IDE we use to operate the Python scripts which communicate with the Arduino and monitors the status of the experiment. The exact versions of the software below are not critical because the programs are relatively simple and have few dependencies.

The following should be installed:

Flycapture SDK: <https://www.flir.com/products/flycapture-sdk/?vertical=machine+vision&segment=iis>

Arduino IDE 2.0.0:

<https://www.arduino.cc/en/software>

JupyterLab or Jupyter Notebook with Python 3 kernel (recommend to install with package manager such as Anaconda/conda):

<https://jupyter.org/install>

Robot control code:

https://github.com/the-rhizodynamics-robot/groot-robot-control.git

**Overview**

The Arduino code is edited on the desktop computer in the Arduino IDE and loaded onto the Arduino. A Python script is run in Jupyterlab on the desktop computer to take user input regarding the light cycle, image save location, etc. Once the user information is set, the Python program will communicate that information to the Arduino, which will employ those run parameters in its operation. In addition, the Python program will continue to run and receive update and error states from the Arduino. If an error state is encountered (the robot has not returned to its home position for an aberrant amount of time, or if the number of images saved on the computer during an imaging cycle is not correct), the Python program sends a warning email to designated recipient addresses. If the error states persist, the robot program is killed and the motor stops moving.

**Getting started**

Clone the robot_control repository from the github link above. The directory will look like this:


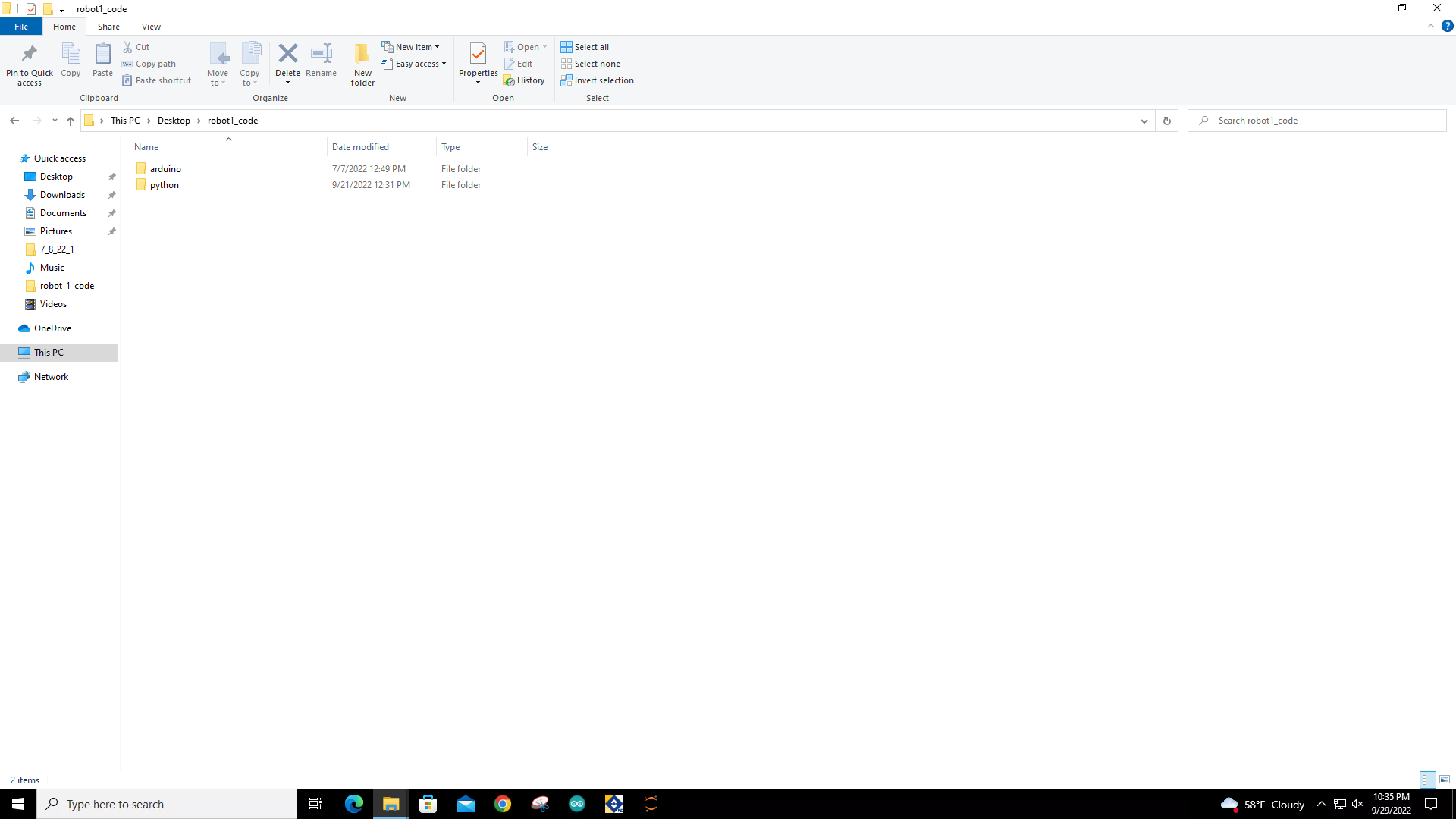


Inside the arduino directory, go into robot_code > and open the .ino file in the Arduino IDE.


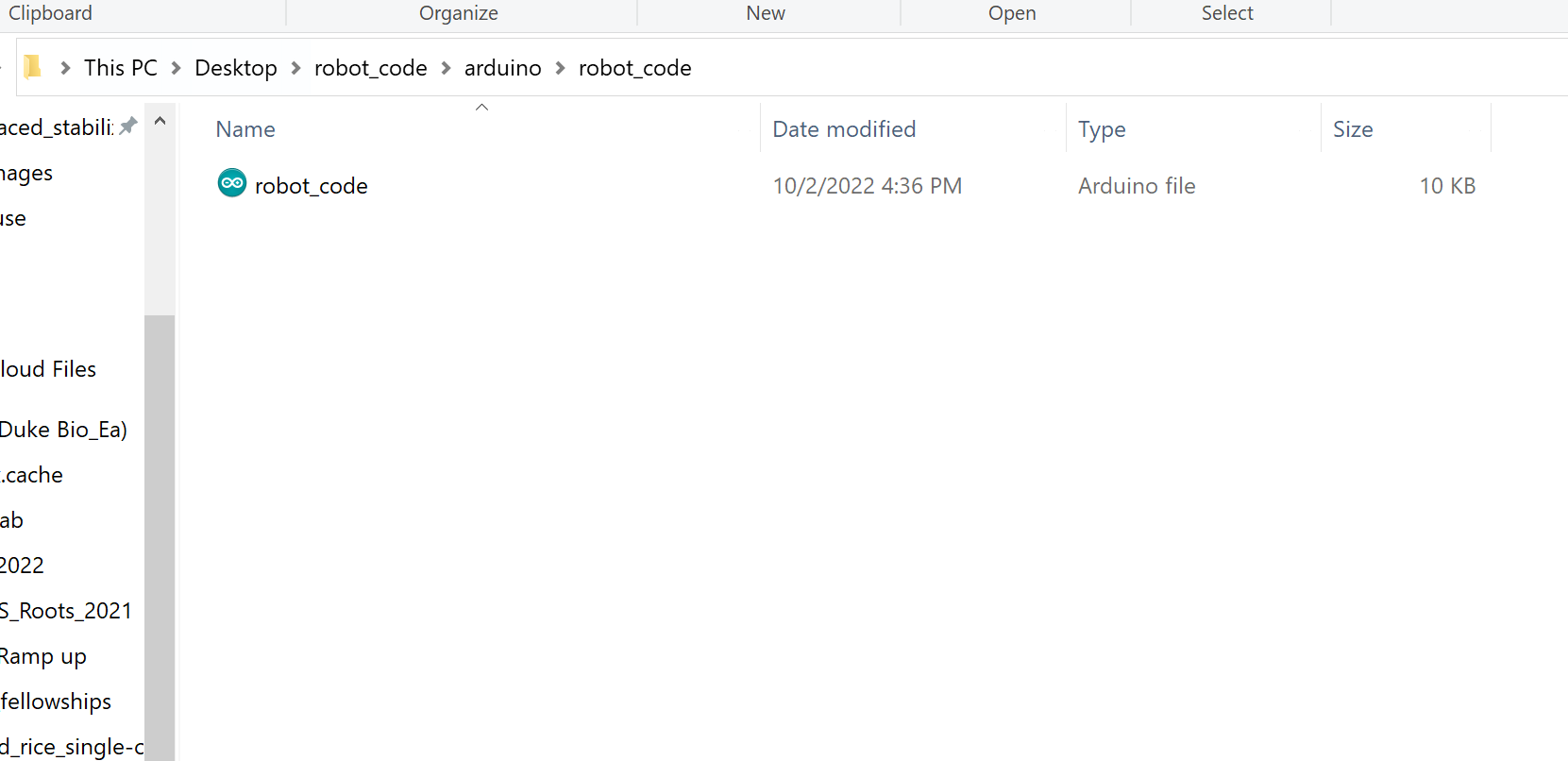


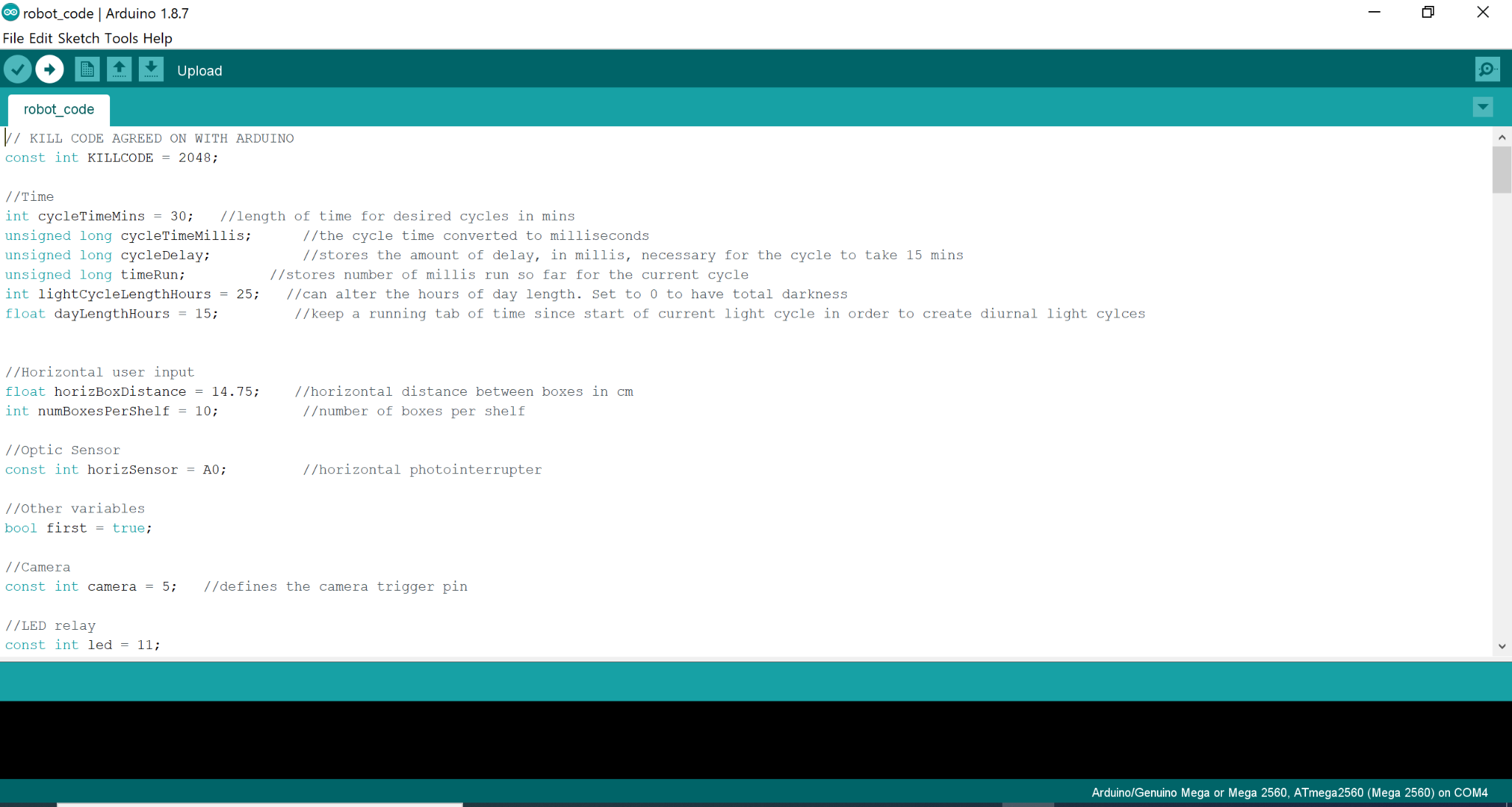


Select Tools then choose your arduino board (we use Mega 2560) as well as the port where it is connected (it will be obvious under the Tools > Port menu).


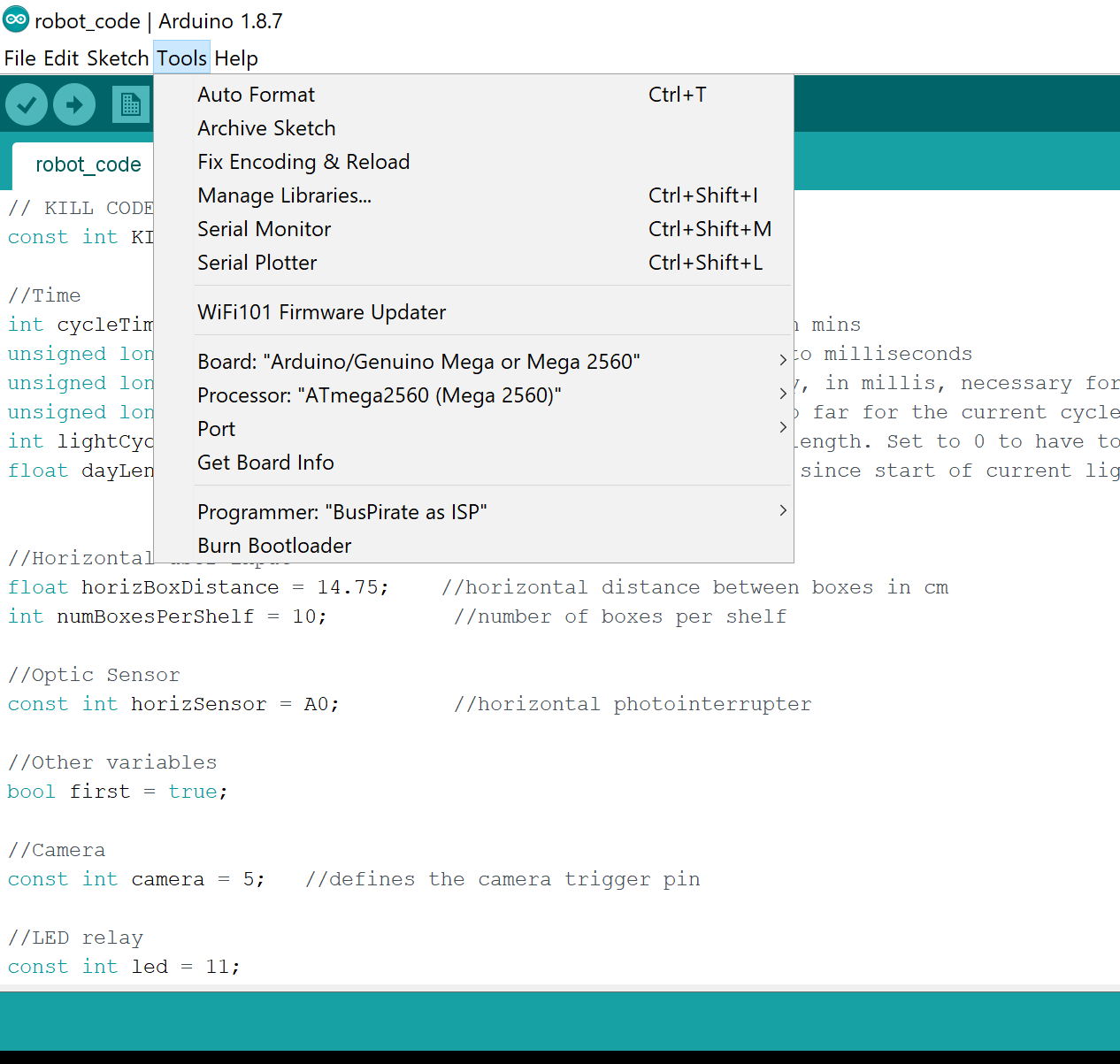


You can check all the pin settings are the ones physically employed on the Arduindo. We are using the pin combinations in the described “One-shelf robot” from Supplement 1.

Load the program onto Arduino by clicking the arrow button.


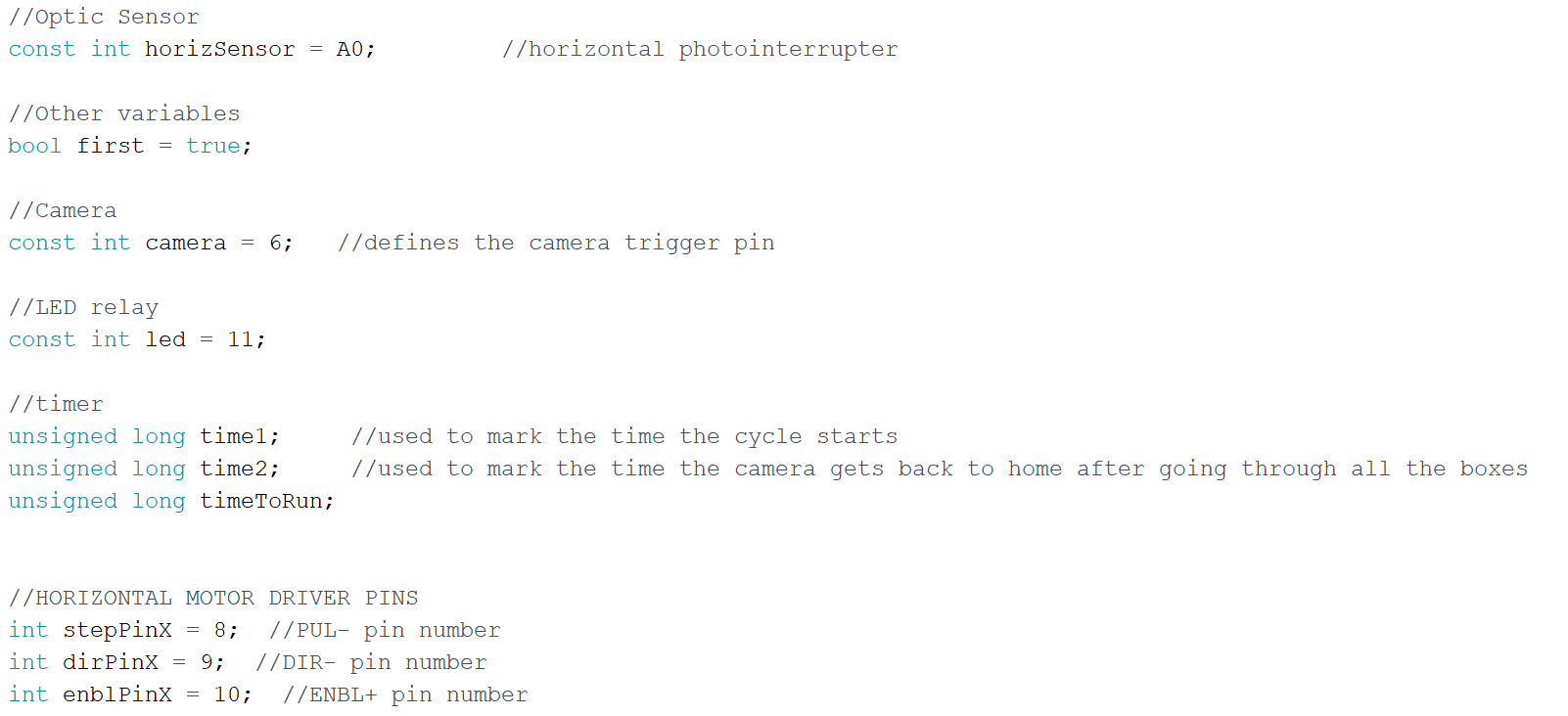


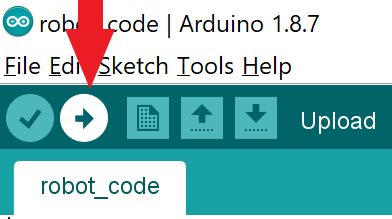


Next start JupyterLab and open the notebook with the “Python” directory of the repository you cloned. You will likely need to install the serial package (pip install pyserial). Otherwise required packages should come preinstalled with Python.

Jupyter notebooks have code broken into cells. Each cell will need to be run in sequence by placing the cursor anywhere inside the cell and clicking the “run” button.


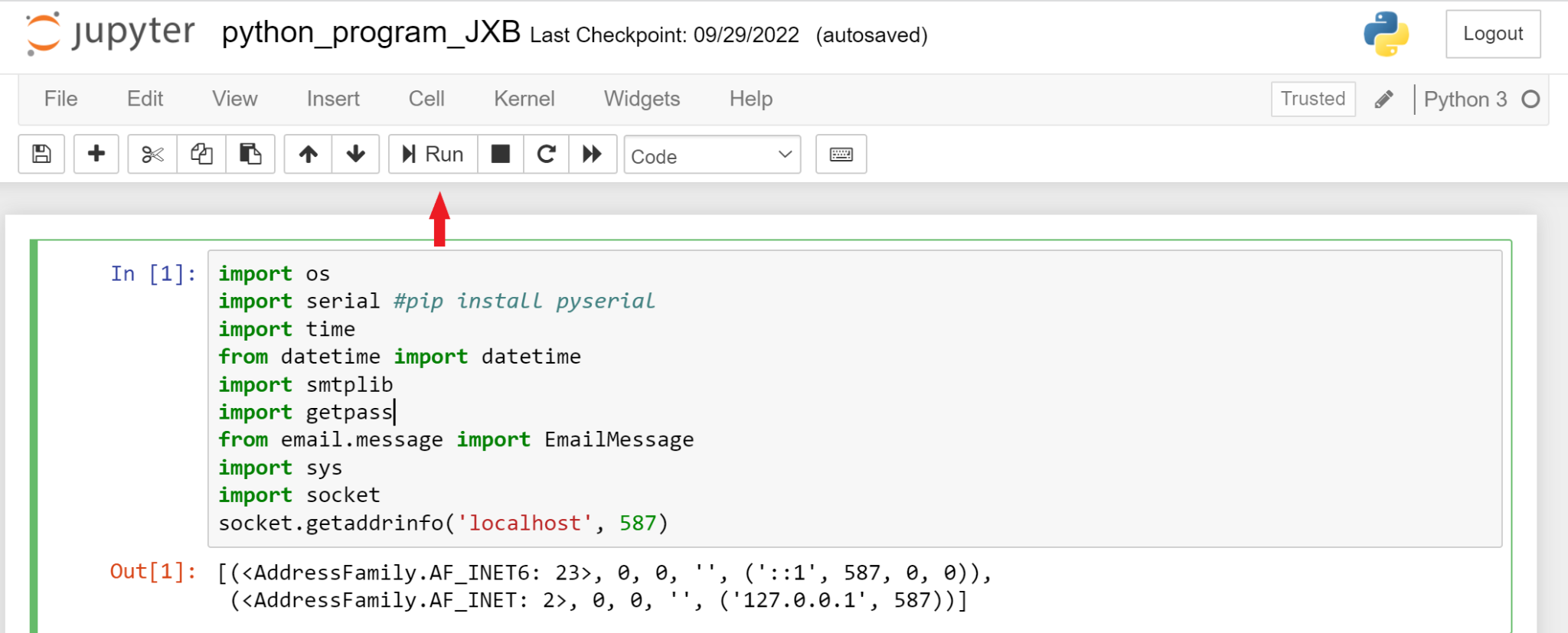


Only the 2nd cell must be modified. Here you edit the “directory” variable to the location you are going to save images in Flycap. Important: We use the convention of naming the directory “MM_DD_YYYY_#” where # refers to the number of shelves being imaged. For this demonstration robot, that number will always be “1.” For larger robots it can be higher. This is used to define the expected number of images taken per cycle, as well ss in sorting the images later on, so it is important. Once you run this cell, the directory “MM_DD_YYYY_#” is created at the “\PATH\TO\” location shown below.

This program also uses an email account to send updates. We use a dummy gmail account for the purpose of sending emails. Any email can be the recipient. Using third-party email software such as this, Google will require you to create an “app password” (ie, a specific password only functional on gmail). This is a useful monitor which relays if there are problems with the robot.


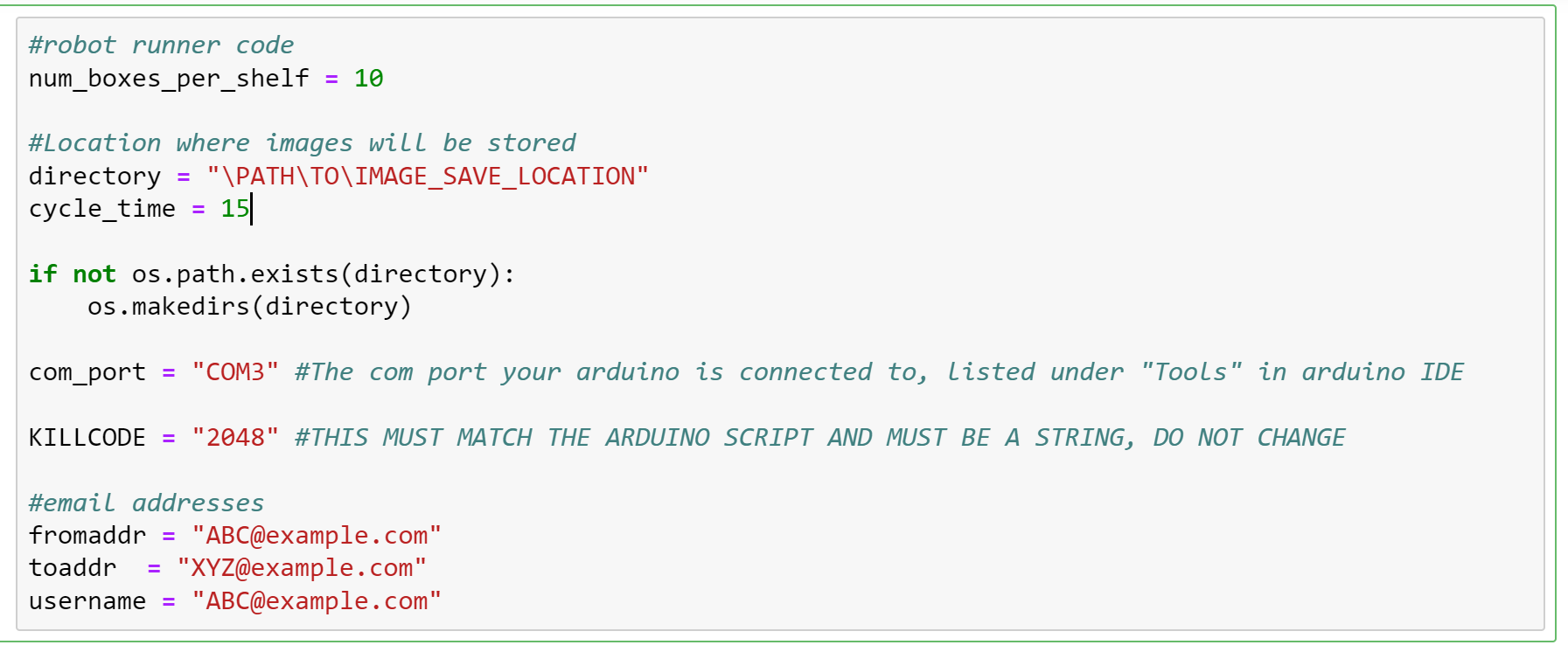


Running the remaining cells will bring up this interaction screen. Enter in the number of shelves (for the small robot it will always be 1), the light cycle, etc. At the end the app will tell you to type “y” after Flycap has been set up (see below):


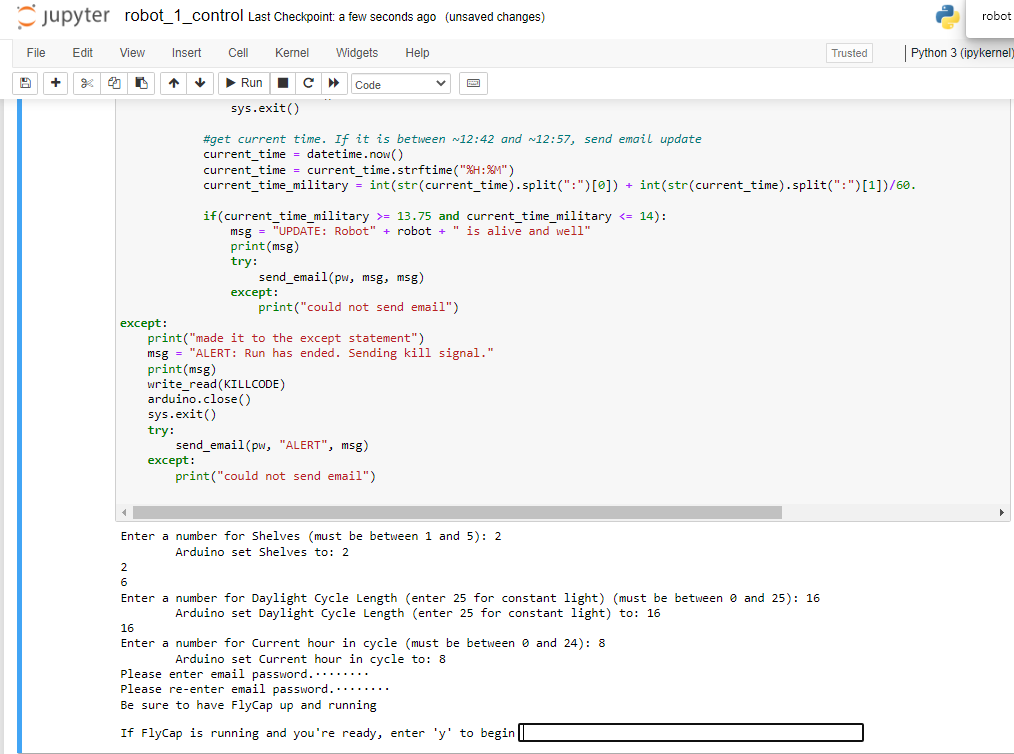


Before clicking “y” above, start Flycap and select the camera.


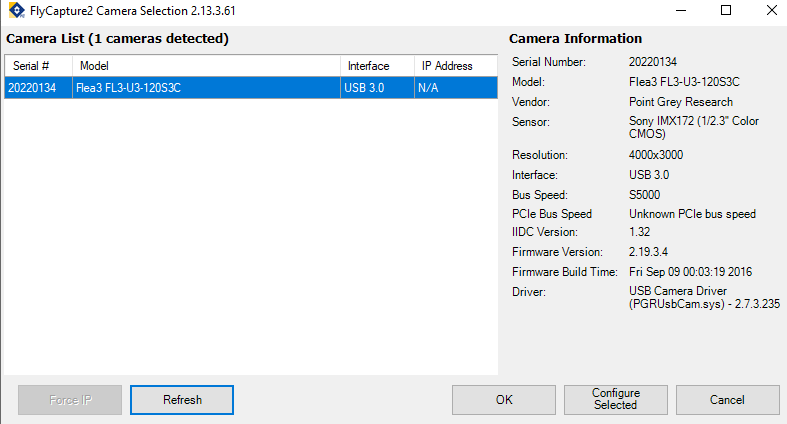


The camera acquisition settings can be altered by clicking Settings > Toggle Camera Control Dialog to bring up the screen below. We typically adjust these once and they are saved thereafter. The “Camera Settings” tab controls things like Exposure etc (you should see a live view which can be used to adjust the lens settings to your desired focal plane, aperture, etc).


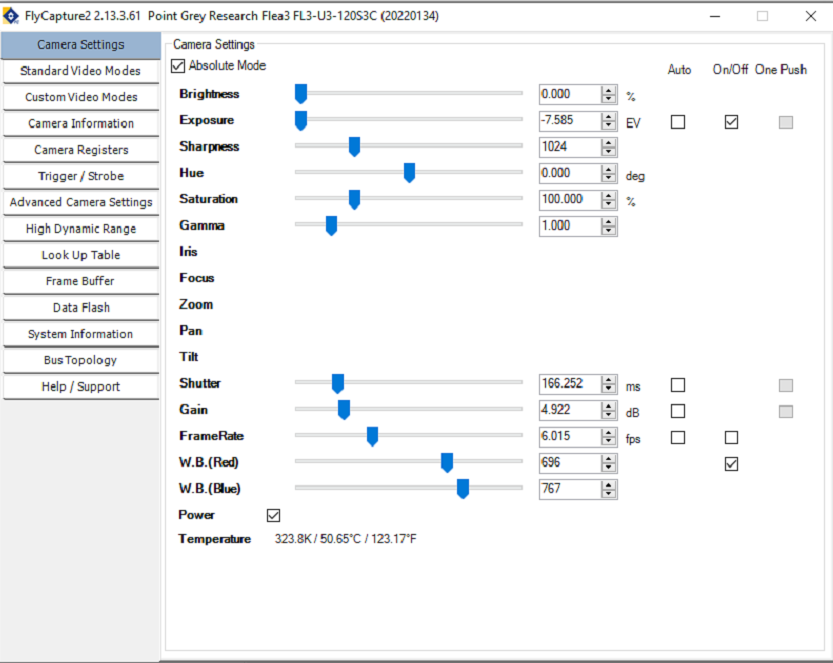


The “Trigger / Strobe” tab should be set the following way in order to trigger image capture by Arduino. Once you select “Enable” trigger, the live view will disappear.


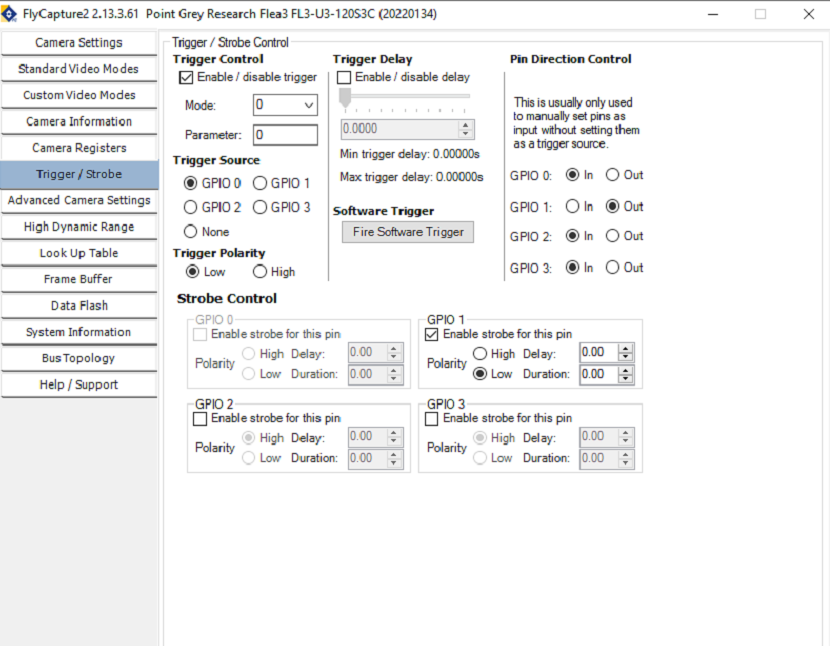


The other tab to look at is the “Custom Video Modes” where you can set the image capture are and the pixel format (we use Raw 8).


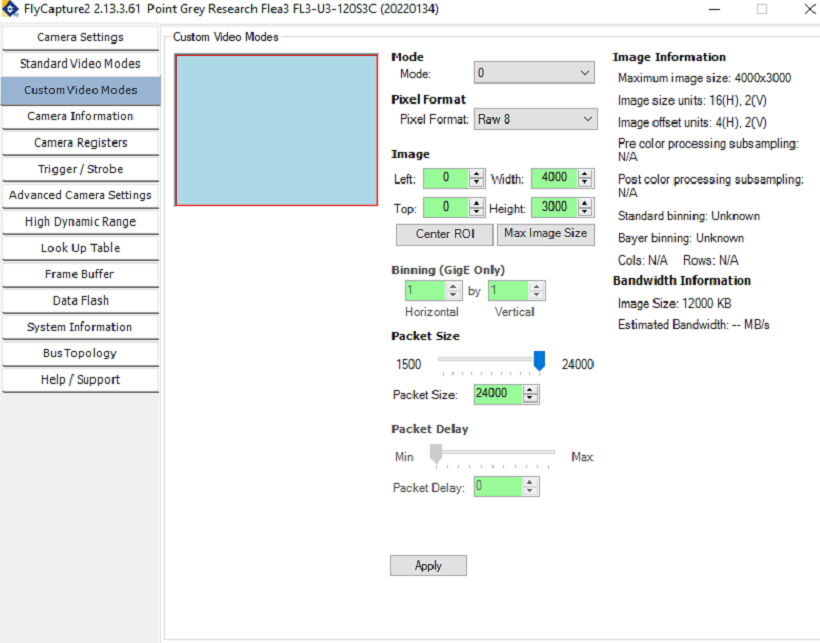


We set up the final acquisition parameters by returning back to the main camer dialog and clciking the “record” button.


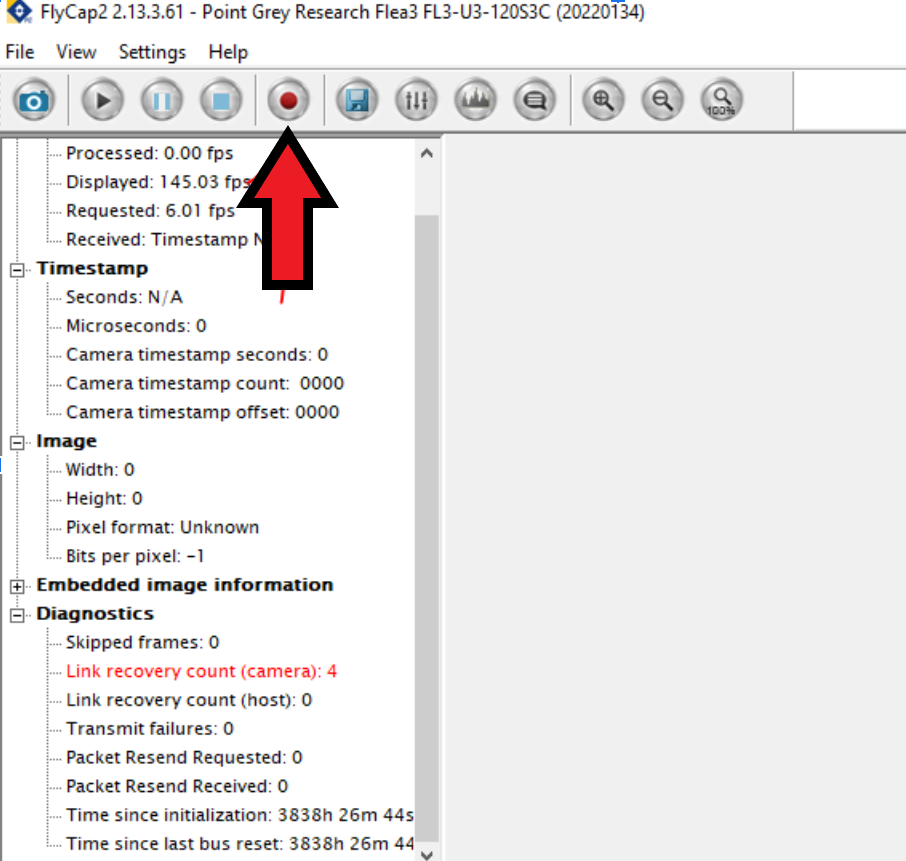


We recommend using the following parameters (PNG with compression level 6 seems good, more compression slows image acquisition and leads to some performance problems). “Capture 0 frames” is an idiosyncratic way for Flycap to indicate you are using a manual trigger. Also here is where you choose the image saving location which you created above in the Python program. We typically use the default prefix (fc2_save), will be appended with sequential image numbers. After you are all done, click “Start Recording” and return to the Jupyter Notebook and type “y” in the final dialog box asking if Flycap has been initialized. This will initialie the robot and begin image aquisition.


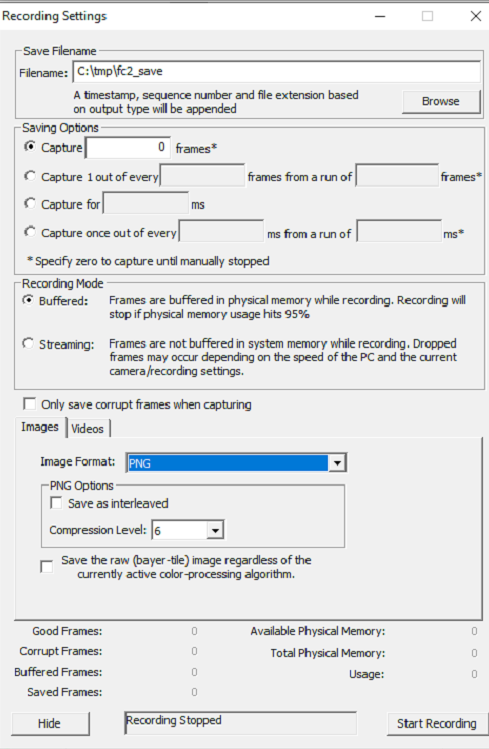

Supplement: S2 File — (DOCX) [file pone.0295823.s002.docx]
