# Supplementary material for: The rhizodynamics robot: Automated imaging system for studying long-term dynamic root growth
Source: PLoS One. 2023 Dec 21;18(12):e0295823. doi: 10.1371/journal.pone.0295823 (PMC10734993; doi:10.1371/journal.pone.0295823)
Supplement: S3 File — (DOCX) [file pone.0295823.s003.docx]

**Supplement 3**

**Experiment numbering and QR label creation code**

We have found the volume of images generated by the GROOT system requires organized management. The raw output of the imaging robot is a directory of images numbered sequentially. The next tasks are to sort these images into directories representing imaging position on the robot (which we refer to as “experiments”), followed by association of each of the experiments with a database of experimental covariates and metadata. While it is technically possible to hand label containers and manually associate each image series with the experimental condition, this quickly becomes unfeasible as experiments scale. To address this issue, we have developed a system where every imaging container is registered in a central spreadsheet with an experiment number as well as with experimental metadata. We then generate a QR code label that encodes this experiment number which is affixed to the container and automatically detected and interpreted by the image sorting program detailed in Supplement 4. Below we detail how to generate the QR code label.

Clone github repository:

https://github.com/the-rhizodynamics-robot/groot-qr-coding.git

It is recommended to use an environment manager such as conda.

We suggest creating a single database of experiments. We use a google spreadsheet where the first two columns contain the experiment number and short description, which are used in the QR labels. The remaining columns are with experimental details


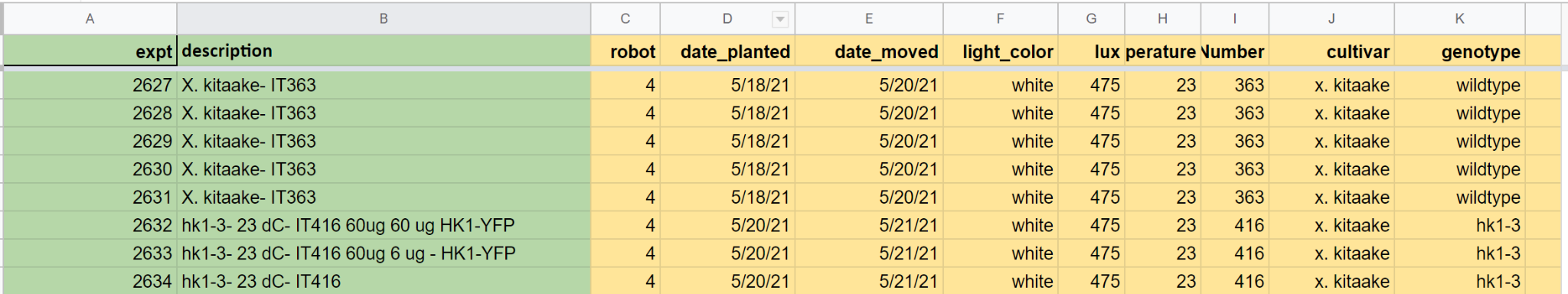


The code detailed in this supplemental will take the first column above (the experiment number) and create a QR code simply encoding this number. It will also concatenate the first and second column (short description) and print it as a human readable reference for easier identification of experimental conditions. An example is provided below:


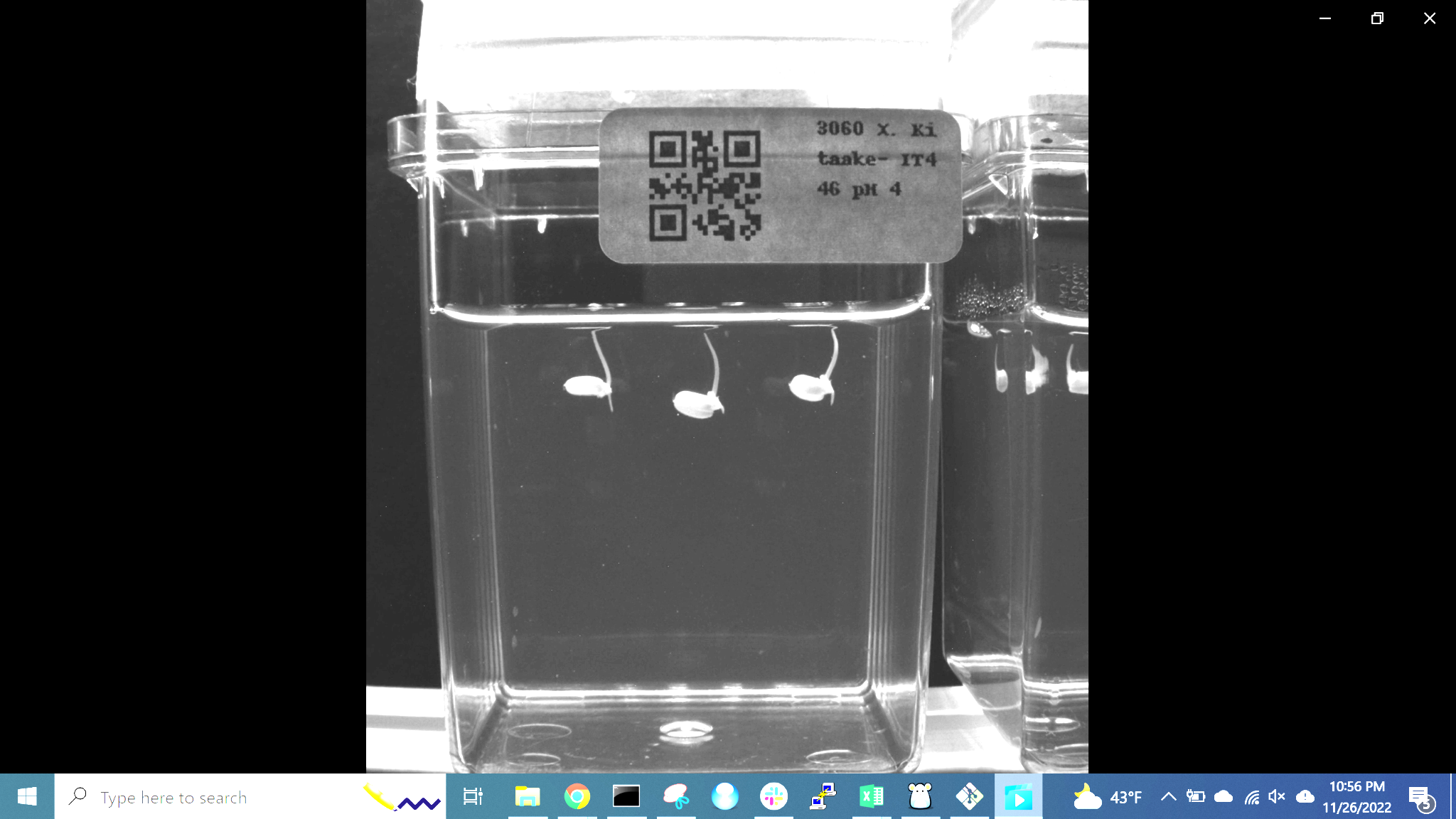


**Use:**

After cloning the repository, there will be two files in the cloned directory (in addition to README): QR_coding.py and labels.csv.


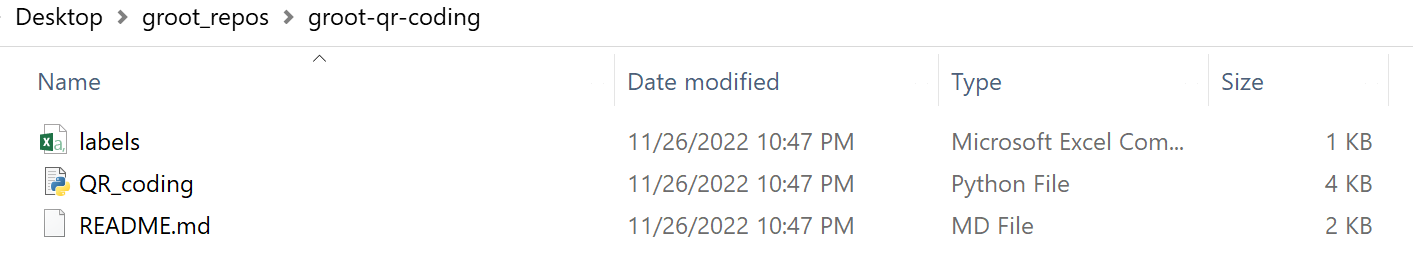


To use the script, simply copy the first 2 columns for the experiments from the experiment spreadsheet into the 2 columns of the labels.csv spreadsheet. Upon cloning, labels.csv will have the following 4 test lines:


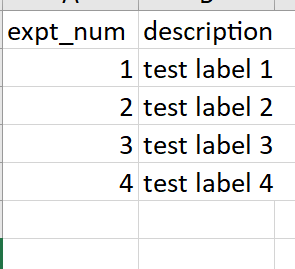


To test the script, you can run python QR_coding.py from the cloned directory. This will read labels.csv which contains the 4 test labels, and should create a PNG file named “1.png” containing 4 test QR codes in the same directory. If you open this image and print onto the labels listed above, you should see the following:


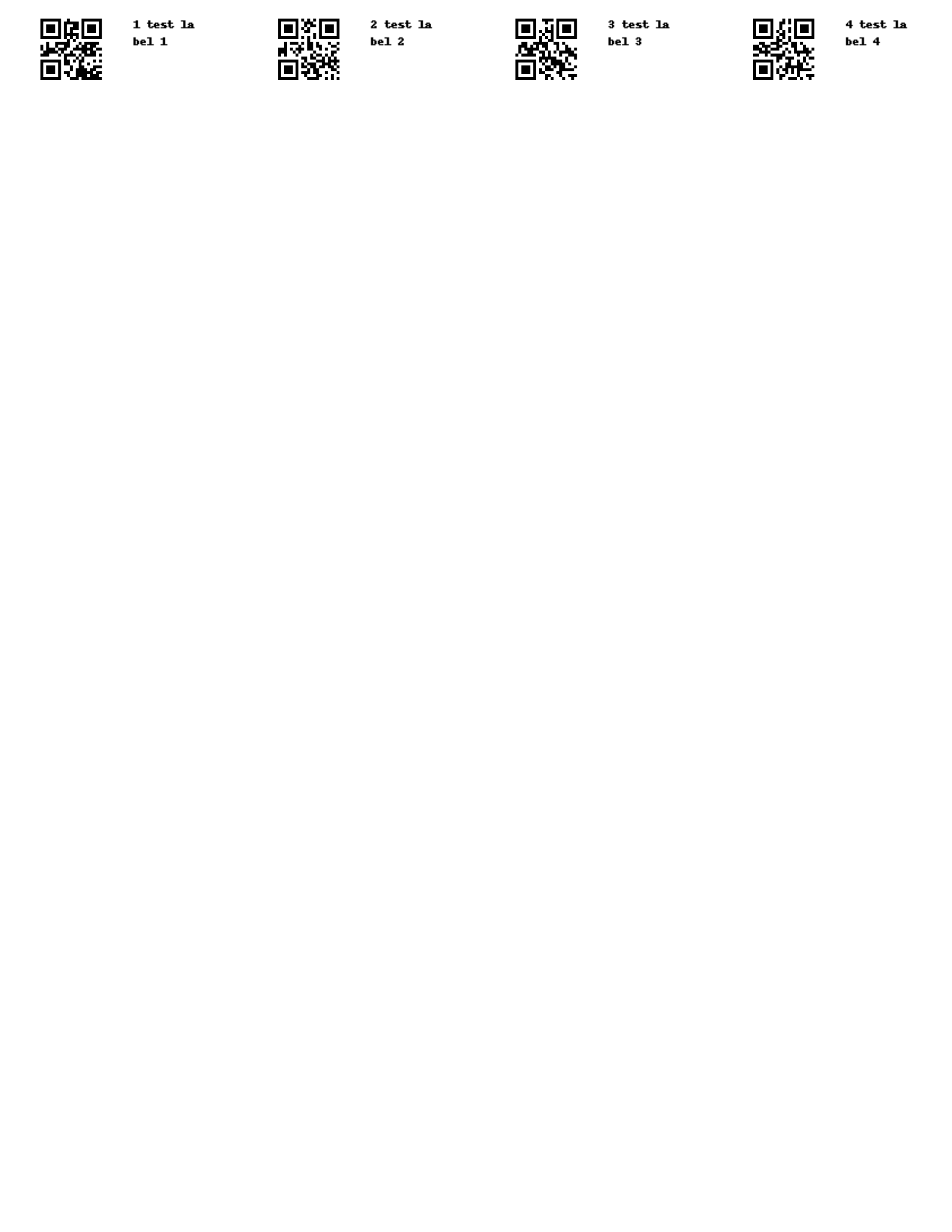


This script will overwrite 1.png so there is no need to remove it before running the script again.

**Troubleshooting:**

We have noticed printing from different image viewing programs can give slightly different offsets and sometimes leads to labels being cut off. This may also happen if using a different printer. If the labels are not lining up properly you can modify the position of the labels on the image. The two places that need to be modified follow these lines:

#Place QR code in appropriate location

#Place description in appropriate location

The code which locates the individual label on the page looks like this:

large[round(40+outer*600):round(540+outer*600), round(170+1400*inner):round(670+1400*inner)] = img

You will most likely need to adjust the numbers highlighted in yellow. These represent the starting offsets in pixels. The upper left corner of the first QR code is placed starting at pixel position (40, 170), and the lower right corner is at position (540, 670). The description is set in a similar manner. The other numbers represent the offset going left to right and top to bottom on the page.
