# Supplementary material for: The rhizodynamics robot: Automated imaging system for studying long-term dynamic root growth
Source: PLoS One. 2023 Dec 21;18(12):e0295823. doi: 10.1371/journal.pone.0295823 (PMC10734993; doi:10.1371/journal.pone.0295823)
Supplement: S4 File — (DOCX) [file pone.0295823.s004.docx]

**Supplement 4**

**Image sorting and movie making**

After images have been acquired with the robot, they are in an unorganized raw format. This supplement describes the installation and running of our custom image sorting and time-lapse movie making pipeline. The output of this pipeline are directories of images for single growth containers named according to the decoded QR code experiment number (and thus match the entries of the experiment spreadsheet described in Supplement 3), as well as time-lapse videos of the experiments. The output of this pipeline can be input to further downstream image analysis pipelines.

We use a Google Cloud virtual machine for image sorting, taking raw images from the robots that have been pushed to a cloud bucket. This system works well for us, but this could also be accomplished on a local machine. We have most recently tested this installation on a Google Cloud N1 instance running Ubuntu 20.04, with 100 GB of disk space and 15 GB RAM. A local machine with similar specs should also function. In the future we hope to containerize this pipeline.

git repository:

https://github.com/the-rhizodynamics-robot/groot-file-sorting.git

**Installation:**

•Clone the repository


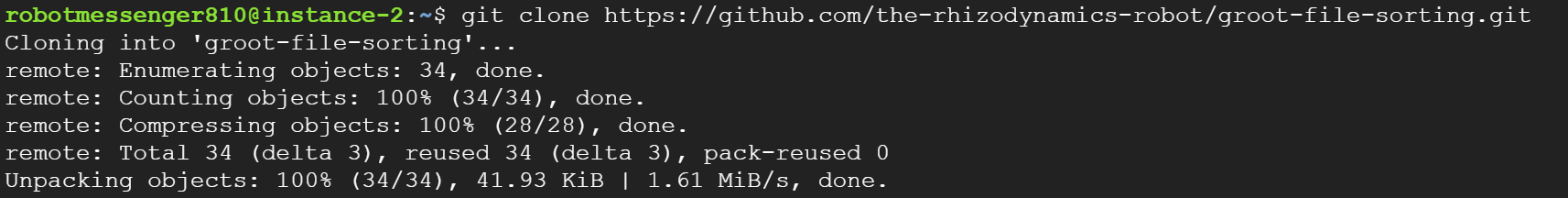


•Install Miniconda (find download link to your OS via google). Download and then run installation bash script.


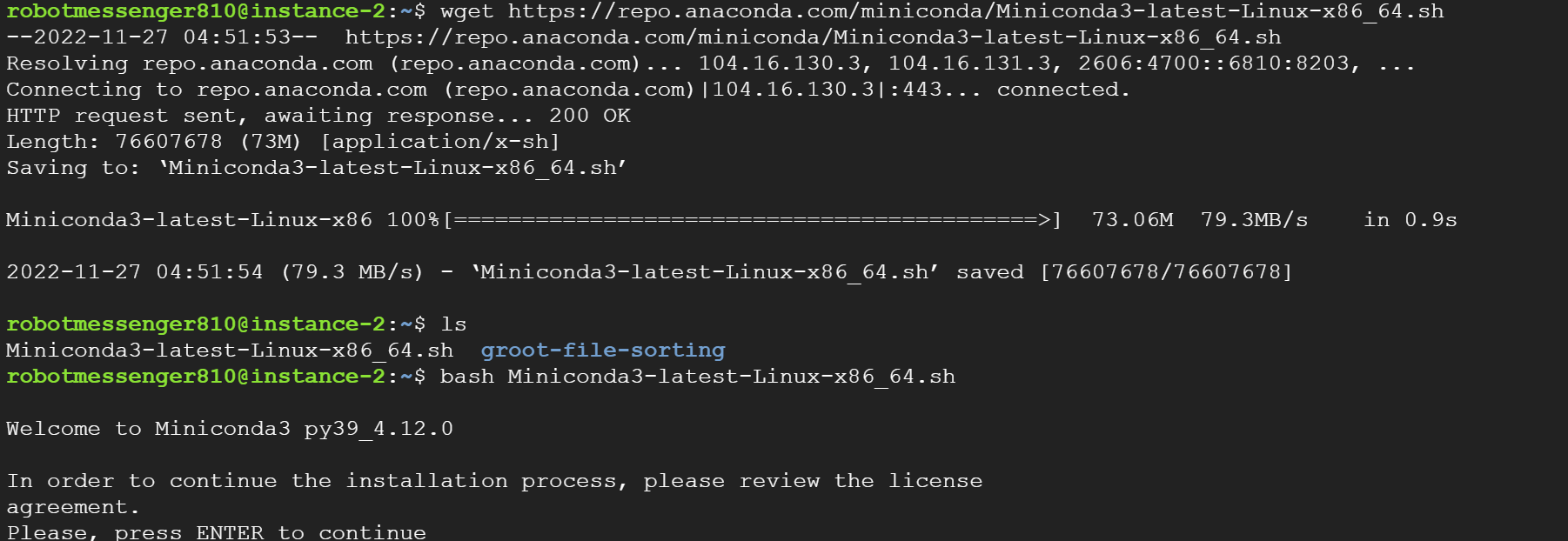


•Create conda env:


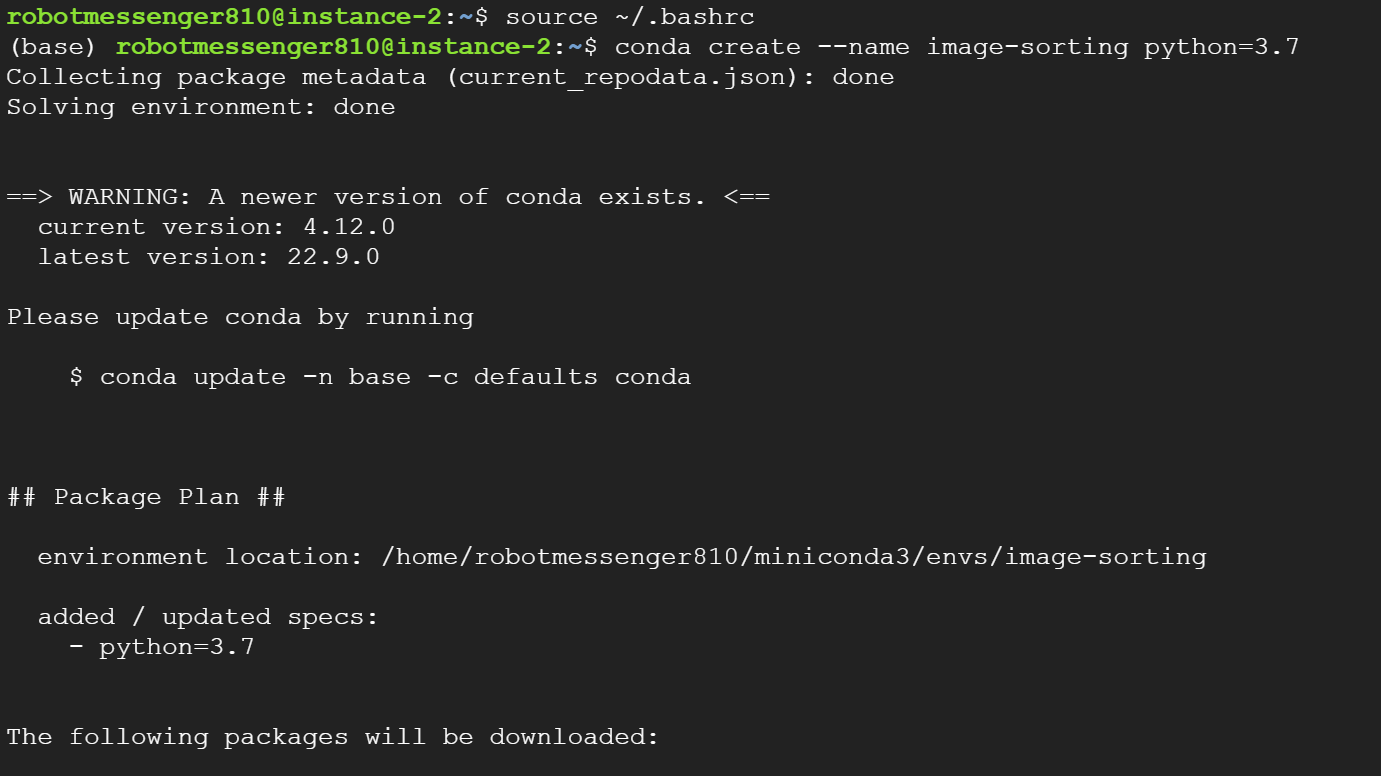


•Activate conda environment:


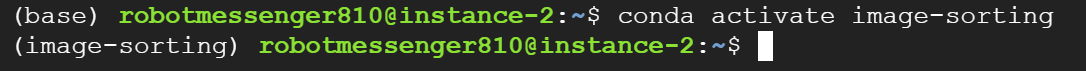


•cd into groot-file-sorting and run:

sudo bash setup.sh


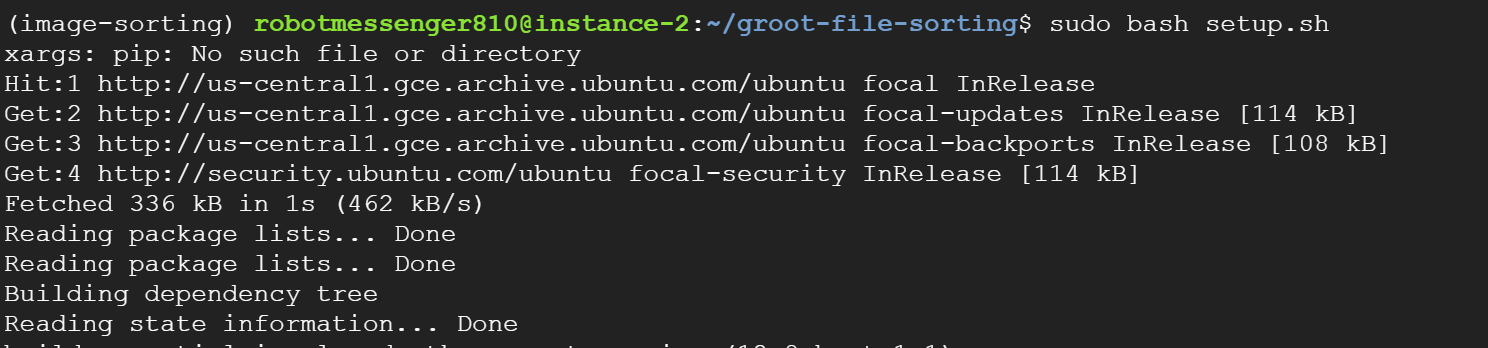


•Inside the cloned repository, there is a “code” and “data” directory.


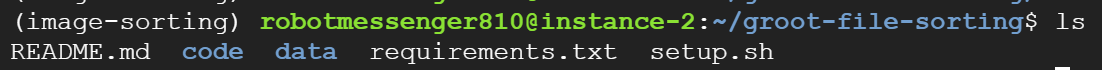


**Running:**

Place zipped, raw images can go into data/unsorted_unlabeled_zipped/ named according to the MM_DD_YYYY_# convention, where # is the number of shelves imaged. This program employs a neural net to identify QR codes in an image series (to differentiate between empty positions on the robot from those with containers).

We recommend running the sorting script from the code/ directory. There are 2 run modes. The first mode takes the oldest zipped file from data/unsorted_unlabelled_zipped, unzips the images into data/robot/master_data/unsorted_unlabeled/, then parses the QR code, and places images into “data/robot/master_data/current_exp/####”, where #### is the experiment number encoded by the QR code. This initial step is accomplished by running:

nohup sh -c 'python robot_image_sorting.py -b 7' & tail -f nohup.out

where the “-b #” flag indicates how many imaging positions there are per shelf (the program needs to know this for the initial image series deconvolution step). The construction of this command allows the terminal connection to close but for the process to complete.

Once the images are in current_exp, they will stay there until the program is run again and a new batch of images is sorted. The program checks if the new batch of images contains QR codes which match those in current_exp. If so, the new images are added to the end of the image series and nothing further happens to that experiment folder. If there is no matching current_exp directory for an image series, then a new current_exp/### directory is created. Finally, if there is one or more current_exp directories which do not have any images added in the incoming batch, then the program assumes that those experiments are completed, and moves the images to “data/robot/master_data/finished_exp”, while also making stabilized mp4 timelapse videos and putting them in data/videos/stabilized. Optionally you can make unstabilized videos placed in data/videos/unstabilized by passing the -d (“do not stabilize”). We constructed the program this way because very frequently only a subset of experiment containers are completed at the time we put additional containers onto the robot. This logic keeps such overlapping experiments organized.

In the second mode, if there are no incoming images, but you would like to process everything in current_exp (for instance, if there are no additional batches incoming for the time being that would “push” the experiments in current_exp to finshed_exp), then you can run the command with the “-t” (transfer) flag:

nohup sh -c 'python robot_image_sorting.py -b 7-t ' & tail -f nohup.out

This will simply move all experiments from current_exp to finished_exp and make stabilized mp4 timelapse videos.

**Troubleshooting:**

On rare occasions, the neural network will find a QR code, but the python function to decode the QR code will fail (usually because the QR code was damaged or creased). In this case, where there is a positive detection of a QR but negative identification, the image series is sent to “data/robot/master_data/junk_review.” The program will not move anything from current_exp to finished_exp. Instead, it will present the message:

“There are # experiment folders that have been sent to junk_review. Please manually move these experiments to the 're_merge' folder in 'junk_review' if you wish to keep them and rename the experiments with the correct experiment number.

PROGRAM ENDING AFTER CURRENT ROBOT RUN”

In this case, you will need to manually rename the directory in junk_review to the correct experiment number, and move to junk_review/remerge. The next time the program is run, it will first check the remerge directory and if there are any image series present, it will move them to current_exp as usual. If connected to a remote server without a graphical interface, your options for checking are either to download a single image and check that way, or the option we use is to install jupyterlab and access from the browser in our local machine. This allows a small amount of graphical interface and file browsing.
