# Supplementary material for: The rhizodynamics robot: Automated imaging system for studying long-term dynamic root growth
Source: PLoS One. 2023 Dec 21;18(12):e0295823. doi: 10.1371/journal.pone.0295823 (PMC10734993; doi:10.1371/journal.pone.0295823)
Supplement: Attachment — Submitted filename: Response to Reviewers.docx [file pone.0295823.s010.docx]

Thank you for the opportunity to submit a revised version of our paper “The rhizodynamics robot: Automated imaging system for studying long-term dynamics,” and for your insightful comments on improving the paper. A detailed summary of our revisions and our responses to the review can be found below in blue.

Reviewer #1: In the paper” The rhizodynamics robot: Automated imaging system for studying long-term root growth” the authors present a low-cost automatic time-lapse imaging system they have created which can be used to study different plant root systems and experimental conditions and allows the acquisition over a long time compared to the systems previously created.

I have read the paper with interest, and I believe that the system is a valuable tool for automated time-lapse acquisitions for prolonged periods of plants of different species, given the flexibility of the system. However, in my opinion, it poses some limitations in root imaging acquisition for several reasons: the image resolution seems poor; therefore, plant species with thin primary roots and lateral roots may be challenging to visualize, and the parameters that can be analyzed may be limited to thick primary roots; temperature control may also be a limitation for certain plant species given the long period of acquisition, especially for growth rate analysis. I believe it is a valuable tool, but to be used for certain types of settings and conditions, which maybe should be pointed out more.

Thank you for the attention to our manuscript. We have addressed the points you mentioned here in the following sections.

Below I noted a few points I suggest for clarification or further improvement of the paper:

1. Please correct in the abstract the misspelling of the name of Oryza sativa and remove the capital letter in the name of the species.

We have corrected this misspelling and the capitalization in the text.

2. Line 191: The authors state that they could analyze the root curvature, root length, and growth rate of the orchid roots. However, the root growth rate is affected by parameters like temperature. From what I understand, the temperature is not controlled, and it may be a system limitation for a prolonged acquisition period for certain plant species. Is there a way in which the system could be improved? You mentioned it briefly in the case of rice acquisition, but I would mention it as one of the improvements or additions that could be made to the system. Would that limit the number of samples that could be analyzed?

This is correct – the system itself does not currently control temperature. The system depicted in Figure 1 is housed in the Atlanta Botanical Garden in a climate-controlled room. To control both temperature and humidity on a smaller scale, the system may be placed inside a growth chamber as mentioned in 146-149. The text was updated to reflect this discussion.

3. Figure 2G; in the Hudsonia montana root growth images, the roots aren’t visible. Could the images be improved or changed? in the caption, remove the capital “M” to the name Hudsonia.

We have zoomed into the images to make the roots more visible. This typo has been corrected.

4. Can the authors improve the quality of the figures presented in the article? The resolution could be better, especially Figure 3.

We have inserted full resolution images into the updated figures. These have been uploaded as separate figures for clarity (Figs 1-3).

5. Is it possible to improve the resolution of the images? or is that in plan for a future work?

To increase the resolution of images, a different camera would need to be used. Our choice of camera was a decision based on cost effectiveness, resolution, and field of view (as it is uncertain where the plant roots will grow relative to the container size). In the case of the orchid imaging, this proved sufficient – however, roots with different sizes and growth speeds may benefit from a different camera. The modular design of this system will allow any camera to integrate with the apparatus with some modifications to the mount and the image acquisition code to reflect the different hardware and software. More uniform lighting conditions would also improve root tracking significantly. The document was updated to reflect this.

We thank the reviewer and editor again for their time.
